# Iron(II) and Manganese(II) Coordination Chemistry Ligated by Coplanar Tridentate Nitrogen-Donor Ligand, 2,6-bis(5-isopropyl-1*H*-pyrazol-3-yl)pyridine

**DOI:** 10.3390/molecules30204128

**Published:** 2025-10-19

**Authors:** Kiyoshi Fujisawa, Yurika Minakawa, David James Young

**Affiliations:** 1 Department of Chemistry, Ibaraki University, Mito 310-8512, Ibaraki, Japan; 2 James Watt School of Engineering, University of Glasgow, University Avenue, Glasgow G12 8QQ, UK; david.j.young@glasgow.ac.uk

**Keywords:** N3-type ligand, coplanar ligand, iron, manganese, crystal structures, physicochemical properties

## Abstract

Coplanar tridentate nitrogen-donor ligands have been extensively employed to stabilize transition metal complexes by chelation. Some complexes exhibit interesting structures and photoluminescent properties. In this work, 2,6-bis(5-isopropyl-1*H*-pyrazole-3-yl)pyridine (denoted as **L**), its iron(II) and manganese(II) dichlorido complexes, and its bis-chelate iron(II) complexes, *viz*. [FeCl_2_(**L**)]·2(MeOH) and [MnCl_2_(**L**)]·2(MeOH), and [Fe(**L**)_2_](PF_6_) ·5(thf), respectively, were synthesized and characterized by single-crystal X-ray structural analysis. These solid-state structures contained N–H donors that formed hydrogen bonds with the coordinated halogenide ions and lattice solvent molecules, methanol or tetrahydrofuran. The iron(II) and manganese(II) dichlorido complexes [FeCl_2_(**L**)]·2(MeOH) and [MnCl_2_(**L**)]·2(MeOH) displayed distorted trigonal pyramidal structures in the solid state. However, [FeCl_2_(**L**)]·2(MeOH) was not stable in methanol and formed the bis-chelate iron(II) complex [Fe(**L**)_2_](FeCl_4_). Therefore, the bis-chelate iron(II) complex [Fe(**L**)_2_](PF_6_)·5(thf) was also synthesized and structurally and spectroscopically authenticated.

## 1. Introduction

Coplanar, tridentate nitrogen-donor ligands have been employed extensively in inorganic and coordination chemistry because of the stability they offer a variety of transition metal complexes by chelation [1]. These ligands impart their thermal stability to the system, and their modification allows for the subtle adjustment of the electronic and steric properties of the resulting metal complexes without significant changes to the coordination environment [2,3,4,5,6,7,8,9,10,11,12,13,14]. These fascinating complexes exhibit interesting structures and photoluminescent properties.

**Figure 1:**
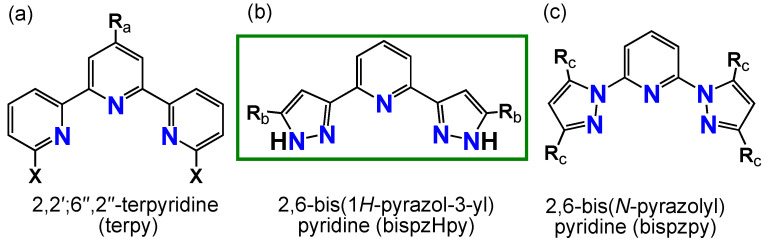
Three types of coplanar tridentate nitrogen-donor ligands, (**a**) 2,2′;6″,2″-terpyridine (terpy), (**b**) 2,6-bis(1*H*-pyrazol-3-yl)pyridine (bispzHpy), and (**c**) 2,6-bis(*N*-pyrazol)pyridine (bispzpy).

One of the most widely studied ligands is 2,2′;6″,2″-terpyridine, *viz.* terpy, with two covalent C_pyridine_—C_pyridine_ bonds (Figure 1a: type a). This popularity is understandable, since terpy is commercially available and straightforward to synthesize and modify at the 4′-position [2,4,5,8,11,12,13]. Another interesting tridentate nitrogen-donor ligand is 2,6-bis(pyrazolyl)pyridine [3,6,9,10,14], which substitutes two pyrazole rings for the two terminal pyridine rings of the terpy structure. This structural variance results in differences in basicity and π-orbital energy, with concomitant differences in kinetic stability, coordination structures, and their properties. In 2,6-bis(pyrazolyl)pyridine ligands, the two pyrazole-based ligands possess either two covalent C_pyrazole_—C_pyridine_ bonds, *viz.* 2,6-bis(1*H*-pyrazol-3-yl)pyridine (bispzHpy) (Figure 1b: type b) [3,6,9,10,14] or two covalent N_pyrazole_—C_pyridine_ bonds, *viz.* 2,6-bis(*N*-pyrazol)pyridine (bispzpy) (Figure 1c: type c) [3,6,9]. These transition metal(II) complexes ligated by these coplanar tridentate nitrogen-donor ligands have been investigated for potential applications in catalysis, supramolecular chemistry, spin-crossover, and luminescent materials [2,3,4,5,6,7,8,9,10,11,12,13,14].

We are interested in the coordination chemistry of tripodal, tridentate nitrogen-donor ligands such as hydrotris(pyrazolyl-1-yl)borate ligands [15,16]. Recently, we explored this chemistry with 2,6-bis(5-isopropyl-1*H*-pyrazol-3-yl)pyridine (bispzHpy, denotes **L**) and 2,6-bis(3,5-diisopropyl-*N*-pyrazol)pyridine (bispzpy, denotes L1) [17,18,19], forming copper(II) complexes with L1 ([CuCl_2_(L1)] and [Cu(OTf)_2_(H_2_O)(L1)]) [17], a zinc(II) complex with L1 ([ZnCl_2_(L1)]) [18], and zinc(II) and copper(II) complexes with **L** ([ZnCl_2_(**L**)], [ZnBr_2_(**L**)], [CuCl_2_(**L**)], and [CuCl(**L**)(thf)](PF_6_)) [19]. In the current work, we report the coordination chemistry of iron(II) and manganese(II) complexes ligated with **L**, structural characterization of these complexes by single X-ray crystallographic measurements, and their IR, far-IR, ^1^H-NMR, and UV-Vis spectroscopic properties.

## 2. Results and Discussion

### 2.1. Synthesis of Complexes

**Figure 2:**
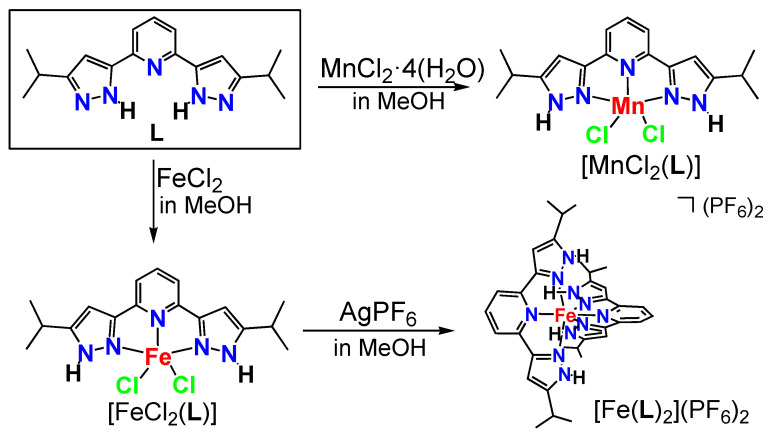
Synthesis of the iron(II) and manganese(II) complexes.

The ligand **L** used herein was synthesized according to a previous report [19]. The iron(II) dichlorido, and manganese(II) dichlorido complexes [FeCl_2_(**L**)]·2(MeOH) and [MnCl_2_(**L**)]·2(MeOH), were obtained by the reaction of **L** with the corresponding metal(II) salts, anhydrous iron(II) chloride (FeCl_2_), and manganese(II) chloride tetrahydrate (MnCl_2_·4H_2_O) in methanol at room temperature (Figure 2). However, an attempt to make a pure complex [FeCl_2_(**L**)] was not successful because of the instability of [FeCl_2_(**L**)]·2(MeOH) in methanol, even at room temperature (*vide infra*). To confirm this dehalogenation reaction, [FeCl_2_(**L**)] was mixed with silver hexafluorophosphate (AgPF_6_) to give the bis-chelate complex [Fe(**L**)_2_](PF_6_)·5(thf). In the free ligand **L**, the N–H group is adjacent to the pyridine ring [19]. On complexation, the H is shifted remotely to the pyridine (*vide infra*).

### 2.2. Solid-State Structures of Iron(II) and Manganese(II) Complexes

**Figure 3:**
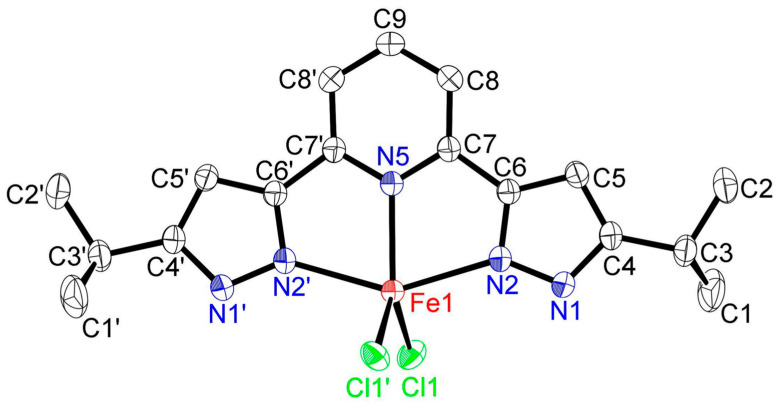
ORTEP view of the iron(II) dichlorido complex [FeCl_2_(**L**)]·2(MeOH) (50% displacement ellipsoids) with the atom-labeling scheme. Hydrogen atoms and methanol molecules of solvates are omitted for clarity. Symmetry operators: −X + 1/2, Y, −Z. Important bond lengths (Å) and angles (°) around iron(II) center: Fe1–Cl1, 2.3207(5); Fe1–N2, 2.247(2); Fe1–N5, 2.129(2); Cl1–Fe1–Cl1′, 114.01(3); Cl1–Fe1–N2, 98.25(4); Cl1–Fe1–N2′, 99.15(4); Cl1–Fe1–N5, 122.997(16); N2–Fe1–N2′, 147.76(7); N2–Fe1–N5, 73.88(5).

The crystal structure of [FeCl_2_(**L**)]·2(MeOH) is illustrated in Figure 3 (50% displacement ellipsoids). This molecule contained two MeOH as crystalline solvates (Figure S1). Its structure is bisected by a mirror plane through Fe, N5, and C9 with symmetry operators: −X + 1/2, Y, −Z. The iron(II) atom is coordinated by the coplanar tridentate nitrogen-donor ligand, **L**, with pentacoordinate geometry involving a Cl_2_N_3_ ligand donor set. The coordination geometry is best described as distorted trigonal bipyramidal. The almost orthogonal, trigonal planes comprise N2, N2′, and N5 and N5, Cl1, and Cl1′. The distortion of the N2—Fe1—N2′ angle from 180°, i.e., 147.76(7)°, is related to the restricted bite angles subtended by the tridentate ligand [N2—Fe1—N5 and N2′—Fe1—N5 = 73.88(5)°]. Another description is provided by the geometric parameter, *τ*_5_, which is calculated from the equation, *τ*_5_ = (*β* − *α*)/60, where *α* and *β* are the largest angles (*β* > *α*) around a five-coordinate metal center: 0.0 for an ideal square pyramidal geometry (*α* = *β* = 180°) and 1.0 for an ideal trigonal bipyramidal geometry (*α* = 180° and *β* = 120°) [20]. In [FeCl_2_(**L**)]·2(MeOH), *τ*_5_ computes to 0.41, a value intermediate between 0.0 (an ideal square pyramidal geometry) and 1.0 (an ideal trigonal bipyramidal geometry) [20]. When compared to [FeCl_2_(**L**)]·2(MeOH), this value (0.41) was almost the same as that of [ZnCl_2_(**L**)]·2(MeOH) (*τ*_5_ = 0.43) and [ZnBr_2_(**L**)]·2(MeOH) (*τ*_5_ = 0.42) and lager than that of [CuCl_2_(**L**)]·3(MeOH) (*τ*_5_ = 0.08) [19]. This value can also be compared with another coplanar tridentate nitrogen-donor ligand of 2,6-bis(3,5-diisopropyl-*N*-pyrazolyl)pyridine (L1); it was clearly larger than those of [CuCl_2_(L1)]·3(MeOH) (*τ*_5_ = 0.26) [17] and [ZnCl_2_(L1)]·2(MeOH) (*τ*_5_ = 0.36) [18]. From this consideration, the structure of [FeCl_2_(**L**)]·2(MeOH) can be confirmed as distorted trigonal bipyramidal coordination geometry. The Fe–N_pz_ (Fe1–N2 and N2′) bond distance is 2.2247 (2) Å and is only somewhat longer than the Fe–N_py_ (Fe1–N5) bond distance (2.129(2) Å). This trend has previously been observed in other complexes with bispzHpy ligands [3,6,9,10,14,19,21,22]. The Fe1–Cl1 distance is 2.3207(5) Å, and the Cl1–Fe1–Cl1′ angle is 114.01(3)°. These bond lengths are almost the same as those observed in [FeCl_2_(L2)] (Type b R_b_ = tBu in Figure 1b, L2 = 2,6-bis(5-tertiary-butyl-1*H*-pyrazol-3-yl)pyridine: Fe–N_pz_: 2.2587(13) Å, Fe–N_py_: 2.130(2) Å, Fe1–Cl1: 2.3097(5) Å, and Cl1–Fe1–Cl1′: 116.44(3)°) [23]. The spin state of [FeCl_2_(L2)] is high-spin using the Evans method [23]. Therefore, from an Fe–N bond length consideration, the spin state of [FeCl_2_(**L**)]·2(MeOH) is also proposed to be high-spin (*vide infra* in NMR section for more evidence).

The crystal packing of [FeCl_2_(**L**)]·2(MeOH) is presented in Figure S2. There are two hydrogen bonds: (i) O–H···Cl’ (O1–H1···Cl1′: 3.130(2) Å, 171.13°, with the symmetry operator −X + 1, −Y + 2, −Z + 1) between the oxygen atom from the crystalline MeOH molecule and the chloride anion, and (ii) N–H···O (N1–H1···O1: 2.758(3) Å, 174.48°, with the symmetry operator −X + 1, −Y + 2, −Z + 1) between the nitrogen from the pyrazolyl ring and the oxygen atom of the crystalline MeOH molecule.

**Figure 4:**
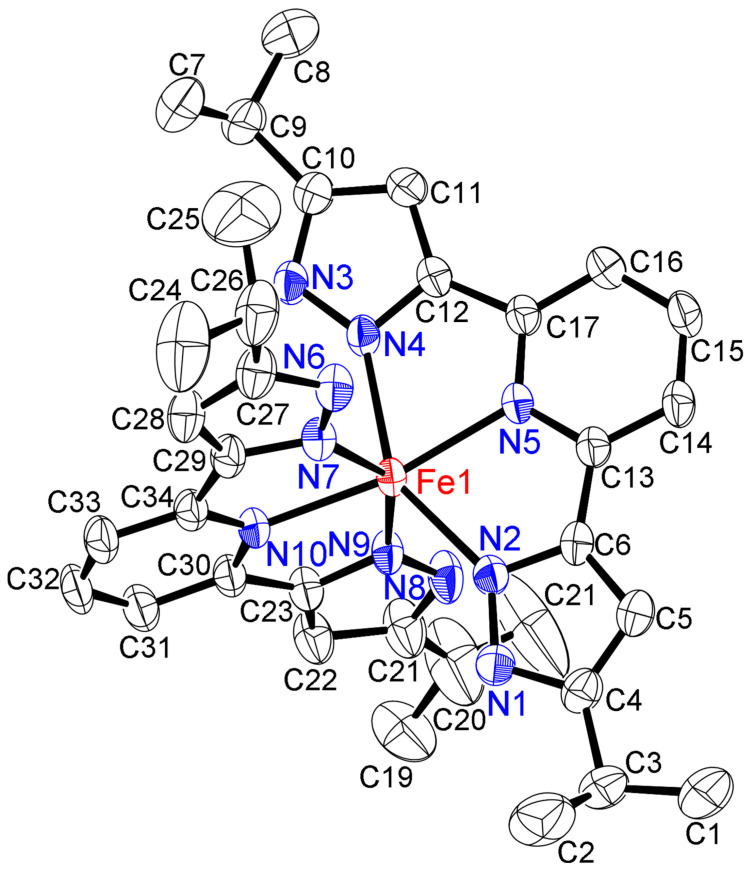
ORTEP view of cation part of the bis-chelate iron(II) complex [Fe(**L**)_2_](PF_6_)_2_·5(thf) (−133 °C) (50% displacement ellipsoids) with the atom-labeling scheme. Hydrogen atoms, counter ions (PF_6_^−^), and thf molecules of solvates are omitted for clarity. Important bond lengths (Å) and angles (°) around iron(II) center: Fe1–N2, 2.185(2); Fe1–N4, 2.212(2); Fe1–N5, 2.138(2); Fe1–N7, 2.203(3); Fe1–N9, 2.175(3); Fe1–N10, 2.138(2); N2–Fe1–N4, 147.75(9); N2–Fe1–N5, 74.19(9); N2–Fe1–N7, 99.91(9); N2–Fe1–N9, 90.37(9); N2–Fe1–N10, 115.29(9); N4–Fe1–N5, 73.56(9); N4–Fe1–N7; 86.74(9); N4–Fe1–N9, 100.95(9); N4–Fe1–N10, 96.90(9); N5–Fe1–N7, 102.13(9); N5–Fe1–N9; 110.56(9); N5–Fe1–N10, 169.93(9); N7–Fe1–N9, 147.28(9); N7–Fe1–N10, 73.63(9); N9–Fe1–N10, 73.89(9).

After the reaction of AgPF_6_ with [FeCl_2_(**L**)]·2(MeOH) in MeOH, the bis-chelate complex [Fe(**L**)_2_](PF_6_)_2_·5(thf) was obtained by recrystallization from thf/heptane. ORTEP diagrams are shown in Figure 4, Figures S3–S5. It is well known that Fe–N bond lengths and some N–Fe–N bond angles are an indication of the spin state of iron(II) complexes, and of the possibility of spin-crossover [24,25,26,27,28,29,30,31,32,33,34,35,36,37,38]. Therefore, solid-state structural analysis was performed at two temperatures, −50 and −133 °C. The results of these low-temperature measurements are given in Table 1, and the results of the higher-temperature measurements (−50 °C) are shown in the SI (Figures S4 and S5). The solid-state structures of [Fe(**L**)_2_](PF_6_)_2_·5(thf) at both low temperatures had the same monoclinic crystal system and *P*2_1_/*c* (#14) space group and contained two PF_6_ counter ions and five thf molecules as crystalline solvates. When measured at −50 °C, the average Fe–N_py_ average was 2.140(3) Å, and the average Fe–N_pz_ average was 2.203(3) Å, consistent with a high-spin state (*vide infra* in NMR section) [9,24,25,26,27,28,29,30,31,32,33,34,35,36,37,38]. X-ray structural analysis was then performed at an even lower temperature of −133 °C. The results showed that the average Fe–N_py_ distance was still 2.138(2) Å, and the average Fe–N_pz_ distance was 2.194(3) Å, indicating that the complex remained in the high-spin state. This result indicates that spin-crossover of [Fe(**L**)_2_](PF_6_)_2_·5(thf) did not occur over this temperature range. Spin-crossover temperatures for known complexes of the [Fe(ligand)_2_]X_2_ type with similar coplanar nitrogen-donor ligands are summarized in Table 1 [26,30,32,33,34,35,36,38] and indicate that an Fe–N bond distance of ~1.9 Å is indicative of low-spin, while an Fe–N bond distance of ~2.1 Å is indicative of high-spin [29]. The coordination geometry was distorted octahedral with six nitrogens of pyrazoles. The above-mentioned average Fe–N_py_ distance (2.138(2) Å) and the average Fe–N_pz_ distance (2.194(3) Å) of [Fe(**L**)_2_](PF_6_)_2_·5(thf) were almost identical to the Fe–N_py_ (2.129(2) Å) and Fe–N_pz_ distance (2.247(2) Å) of [FeCl_2_(**L**)]·2(MeOH), indicating no influence on mono- and bis-chelating of the ligand **L**.

The crystal structure of [Fe(**L**)_2_](PF_6_)_2_·5(thf) at −133 °C contained hydrogen bonds between the N–H and thf solvates, but no intermolecular hydrogen bonds were evident, as observed for other complexes (Figures S3 and S4). The relevant bond distances were N–H···O (N1–H1···O1: 2.747(5) Å, 165.51°; N3–H3···O2: 2.770(4) Å, 173.93°; N6–H6···O3: 2.739(4) Å, 177.03°; and N8–H8···O4: 2.757(4) Å, 163.05° between the nitrogen atom from the pyrazolyl ring and the oxygen atom of the crystalline thf molecule. Another crystalline thf molecule exists independently without any hydrogen bonds and fills crystallographic voids to stabilize the crystal structure.

**Figure 5:**
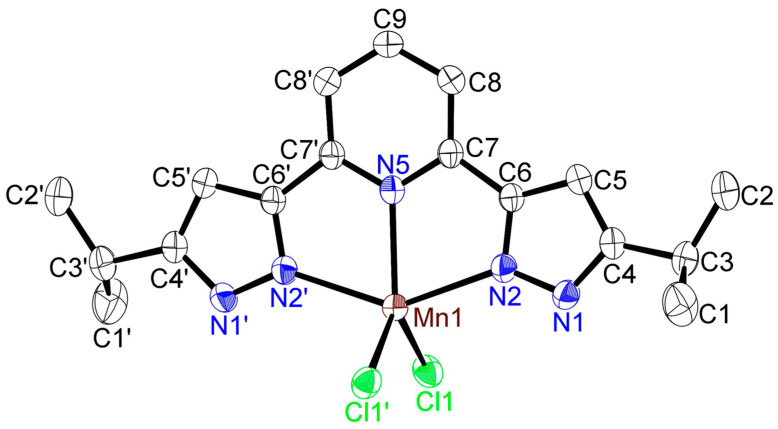
ORTEP view of the manganese(II) dichlorido complex [MnCl_2_(**L**)]·2(MeOH) (50% displacement ellipsoids) with the atom-labeling scheme. Hydrogen atoms and methanol molecules of solvates are omitted for clarity. Symmetry operators: −X + 1/2, Y, −Z. Important bond lengths (Å) and angles (°) around iron(II) center: Mn1–Cl1, 2.3777(4); Mn1–N2, 2.2929(18); Mn1–N5, 2.226(2); Cl1–Mn1–Cl1′, 112.37(2); Cl1–Mn1–N2, 100.85(4); Cl1–Mn1–N2′, 99.39(4); Cl1–Mn1–N5, 123.814(12); N2–Mn1–N2′, 143.19(6); N2–Mn1–N5, 71.59(4).

The ORTEP view of [MnCl_2_(**L**)]·2(MeOH) is illustrated in Figure 5 (50% displacement ellipsoids). This molecule contained two MeOH molecules as crystalline solvates (Figure S6). Its structure is bisected by a mirror plane through Mn, N5, and C9 with symmetry operators: −X + 1/2, Y, −Z. The manganese(II) atom is coordinated by the coplanar tridentate nitrogen-donor ligand, **L**, with pentacoordinate geometry involving the Cl_2_N_3_ ligand donor set as with [FeCl_2_(**L**)]·2(MeOH) (Figure 3). This coordination geometry is best described as distorted trigonal bipyramidal. The trigonal planes compromise N2, N2′, and N5 and N5, Cl1, and Cl1′. These planes were almost perpendicular. The distortion of the N2—Mn1—N2′ angle from 180°, i.e., 143.19(6)°, is related to the restricted bite angles subtended by the tridentate ligand [N2—Mn1—N5 and N2′—Mn1—N5 = 71.59(4)]. In [MnCl_2_(**L**)]·2(MeOH), the value of *τ*_5_ is 0.32, a value intermediate between 0.0, an ideal square pyramidal geometry and 1.0, and an ideal trigonal bipyramidal geometry [20]. When compared to other complexes with **L**, this value (0.32) was smaller than that of [FeCl_2_(**L**)]·2(MeOH) (*τ*_5_ = 0.41), [ZnCl_2_(**L**)]·2(MeOH) (*τ*_5_ = 0.43) and [ZnBr_2_(**L**)]·2(MeOH) (*τ*_5_ = 0.42) and larger than that of [CuCl_2_(**L**)]·3(MeOH) (*τ*_5_ = 0.08) [19]. Therefore, [MnCl_2_(**L**)]·2(MeOH) also can also be described as having distorted trigonal bipyramidal coordination geometry. The Mn–N_pz_ (Mn1–N2 and N2′) bond distance is 2.2929(18) Å and only somewhat longer than the Mn–N_py_ (Mn1–N5) bond distance (2.226(2) Å). This trend has previously been observed in other complexes with bispzHpy ligands [10,14,19,21,22]. The Mn1–Cl1 distance is 2.3777(4) Å, and the Cl1–Mn1–Cl1′ angle is 112.37(2)°. These values are slightly different from those of other Fe(II), Zn(II), and Cu(II) complexes, viz. the Fe1–Cl1 distance (2.3207(5) Å) and Cl1–Fe1–Cl1′ angle (114.01(3)°) in [FeCl_2_(**L**)]·2(MeOH); the Zn–Cl distance (2.2655(4) Å) and Cl–Zn–Cl’ angle (114.54(2)°) in [ZnCl_2_(**L**)]·2(MeOH); and the Cu–Cl1 (2.5963(8) Å), Cu–Cl2 (2.2096(8) Å), and Cl1–Cu–Cl2 angle (108.18(3)°) in [CuCl_2_(**L**)]·2(MeOH), correlating with their different ionic radii [18]. With the exception of [CuCl_2_(**L**)]·2(MeOH), complexes of type [MCl_2_(**L**)]·2(MeOH) where M = Mn, Fe, and Zn have the same distorted trigonal bipyramidal geometry, and *d* (Mn–Cl) > *d* (Fe–Cl), *d* (Zn–Cl) is valid, indicating an Irving–Williams series [39,40,41].

The molecular packing of [MnCl_2_(**L**)]·2(MeOH) is presented in Figure S7. Two hydrogen bonds, (i) O–H···Cl’ (O1–H1D···Cl1′: 3.131(2) Å, 167.08°, with the symmetry operator X, −Y + 1, Z + 1) between the oxygen atom from the crystalline MeOH molecule and the chloride anion, and (ii) N–H···O (N1–H1···O1: 2.772(1) Å, 172.42° between the nitrogen atom from the pyrazolyl ring and the oxygen atom of the crystalline MeOH molecule, are observed.

### 2.3. Infrared and Far-Infrared Spectroscopy

IR spectra for the free ligand **L**, [FeCl_2_(**L**)]·2(MeOH), [Fe(**L**)_2_](PF_6_)_2_·5(thf), and [MnCl_2_(**L**)]·2(MeOH) are reproduced in Figure S8 and listed with their assignments in the experimental section. Raman spectra for the colorless ligand **L** and the manganese(II) dichlorido complex [MnCl_2_(**L**)]·2(MeOH) are shown in Figure S9. Spectral assignments were made with reference to the corresponding spectra of [ZnCl_2_(**L**)]·2(MeOH) [19]. The N–H stretching of this ligand **L** at 3214 cm^−1^ undergoes a clear shift on complexation to 3182 cm^−1^ for the iron (II) dichlorido complex [FeCl_2_(**L**)]·2(MeOH), to 3371 cm^−1^ for the iron(II) bis-chelate complex [Fe(**L**)_2_](PF_6_)_2_·5(thf), and to 3167 cm^−1^ for the manganese(II) dichlorido complex [MnCl_2_(**L**)]·2(MeOH), indicating that the N–H remain attached at the pyrazole ring. The broad signal around the 3300–3200 cm^−1^ region is likely due to intramolecular hydrogen bonding. In addition, broad O–H stretching bands were also observed around 3400–3600 cm^−1^. The characteristic C–H stretching modes of the ring residues appear in the 3100–3000 cm^−1^ region. By comparison, the C–H stretching vibrations of the isopropyl residue appear below 3000 cm^−1^. The P–F stretching for [Fe(**L**)_2_](PF_6_)_2_·5(thf) was evident at 847 and 557 cm^−1^ [19].

**Figure 6:**
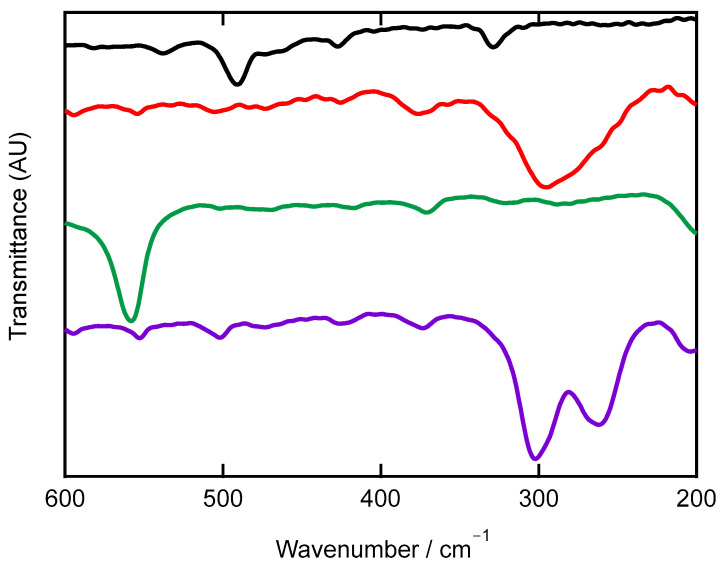
Far-IR spectra in the range from 600 to 200 cm^−1^ (CsI pellets) of the ligand **L** (black trace), [FeCl_2_(**L**)]·2(MeOH) (red trace), [Fe(**L**)_2_](PF_6_)_2_·5(thf) (green trace), and [MnCl_2_(**L**)]·2(MeOH) (purple trace).

In the far-IR region, the characteristic stretching frequencies of M–Cl (M = Fe and Mn) were observed as shown in Figure 6. The most intense bands appeared at 295 (broad) and 261 (broad and shoulder) cm^−1^ for the iron (II) dichlorido complex [FeCl_2_(**L**)]·2(MeOH) and at 302 and 262 cm^−1^ for the manganese(II) dichlorido complex [MnCl_2_(**L**)]·2(MeOH). The order of M–Cl stretching (M = Mn, Fe, and Zn) did not correlate with the relative solid-state bond distance lengths, reflecting differences in their coordination structures. The M–N stretching vibrations can be tentatively assigned as [FeCl_2_(**L**)] *ν*(Fe–N) 650 cm^−1^, [MnCl_2_(**L**)] *ν*(Mn–N) 668 cm^−1^, [Fe(**L**)_2_](PF_6_) *ν*(Fe–N) 651 cm^−1^ and [CuCl_2_(**L**)] *ν*(Cu–N) 647 cm^−1^ [19], [ZnCl_2_(**L**)] *ν*(Zn–N 671) cm^−1^ [19], [ZnBr_2_(**L**)] *ν*(Zn–N 671) cm^−1^ [19], and [CuCl(**L**)(thf)](PF_6_) *ν*(Cu–N) 654 cm^−1^ [19]. The order is *ν*(Zn–N) 671 cm^−1^ in [ZnCl_2_(**L**)] and [ZnBr_2_(**L**)] > *ν*(Mn–N) 668 cm^−1^ in [MnCl_2_(**L**)] > *ν*(Fe–N) 650 cm^−1^ in [FeCl_2_(**L**)], consistent with an Irving–Williams series [39,40,41]. The Raman spectral features of [MnCl_2_(**L**)]·2(MeOH) were shifted relative to the free ligand, **L** (Figure S9).

### 2.4. NMR Spectroscopy

**Figure 7:**
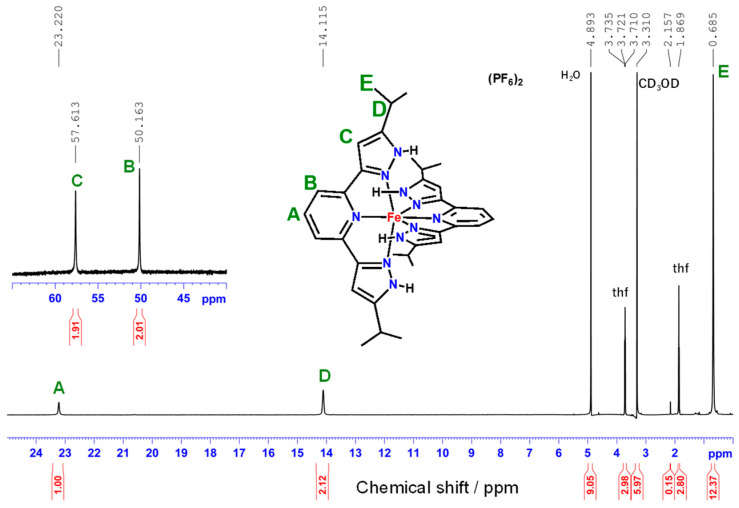
^1^H-NMR spectrum of [Fe(**L**)_2_](PF_6_)_2_·5(thf) in CD_3_OD at room temperature.

The ^1^H-NMR spectra of the two iron complexes [FeCl_2_(**L**)]·2(MeOH) and [Fe(**L**)_2_](PF_6_)_2_·5(thf) contained peaks spread over a wide range from 60 ppm to 0 ppm (Figure 7, Figures S10 and S11), consistent with paramagnetic high-spin iron(II) complexes [40].

The ^1^H-NMR spectrum of [FeCl_2_(**L**)]·2(MeOH) showed two types of resonances (Figure S10). However, when stored at room temperature for several days, spectral changes were observed, with a decrease in one pair of signals and an increase in another pair of signals (Figures S11 and S12). From these spectral changes, we hypothesized that [FeCl_2_(**L**)]·2(MeOH) transformed into another complex over time. The ^1^H-NMR spectrum of [Fe(**L**)_2_](PF_6_)_2_·5(thf) was also acquired (Figure 7). Comparing the spectrum of the product of [FeCl_2_(**L**)]·2(MeOH) in CD_3_OD (Figure S10) with that of [Fe(**L**)_2_](PF_6_)_2_·5(thf) (Figure 7) revealed striking similarities. Therefore, the product of [FeCl_2_(**L**)]·2(MeOH) in CD_3_OD is likely the bis-chelate iron(II) complex [Fe(**L**)_2_](FeCl_4_). In scientific reports of iron(II) complexes of coplanar tridentate nitrogen-donor ligands, the formation of bis-chelate iron(II) complexes in polar solvents such as thf was previously noted [27,42].

X-ray structural analysis revealed that the solvate thf is strongly hydrogen-bonded to the N–H group. The peaks corresponding to thf could not be completely removed during drying *in vacuo* and were observed at 3.72 and 1.87 ppm. The time-dependent ^1^H-NMR spectral changes are presented in Figure S12. Initially, we observed the ratio of [FeCl_2_(**L**)]: [Fe(**L**)_2_](FeCl_4_) to be 1:2.6, but after 48 h, the ratio changed to 1:0.9. Therefore, this dimerization reaction was slow and did not proceed to completion under these experimental conditions.

### 2.5. UV-Vis Spectroscopy

We employed UV-Vis spectroscopy to better understand this transformation of the bis-chelate complex. The UV-Vis spectra of [FeCl_2_(**L**)]·2(MeOH) and [Fe(**L**)_2_](PF_6_)_2_·5(thf) in the MeOH solution were acquired and were almost identical (Figure S13). Therefore, the complex obtained from dissolving [FeCl_2_(**L**)]·2(MeOH) in the MeOH solution was confirmed as [Fe(**L**)_2_]^2+^. Both spectra contained broad signals for π-π* and n-π* transitions originating from **L** at 315 and 253 nm for [Fe(**L**)_2_](FeCl_4_) and 316 and 252 nm for [Fe(**L**)_2_](PF_6_)_2_·5(thf), which were slightly red-shifted from **L** at 307 and 242 nm [19]. The same bands for [MnCl_2_(**L**)]·2(MeOH) were also overserved at 317 and 255 nm.

**Figure 8:**
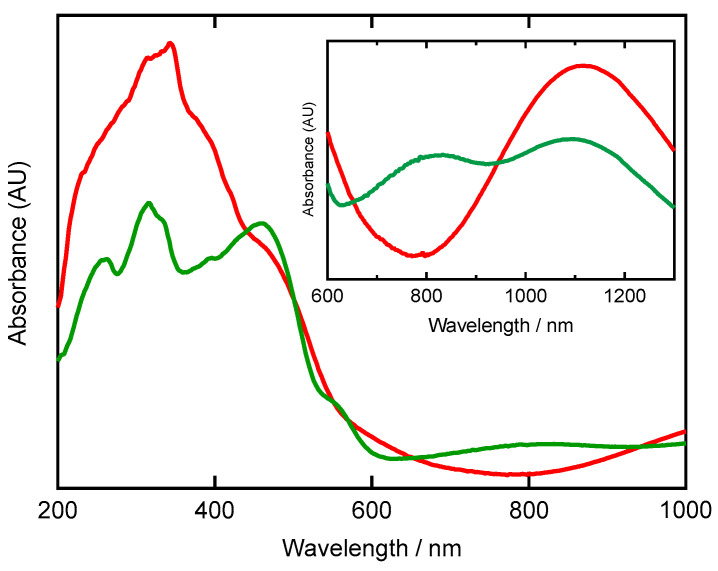
Diffuse reflectance spectra of [FeCl_2_(**L**)]·2(MeOH) (red trace) and [Fe(**L**)_2_](PF_6_)_2_·5(thf) (green trace) at room temperature.

Diffuse reflectance spectra in the solid state of [FeCl_2_(**L**)]·2(MeOH) and [Fe(**L**)_2_](PF_6_)_2_·5(thf) (Figure 8) revealed significant differences in peak shapes between these complexes. The π-π* and n-π* transitions were very broad. Additionally, above 800 nm, one broad peak was observed at 1116 nm for [FeCl_2_(**L**)]·2(MeOH) and two broad peaks were observed at 826 and 1098 nm for [Fe(**L**)_2_](PF_6_)_2_·5(thf). The d-d transition bands of six-coordinate iron(II) complexes appear around 1000 nm [43].

## 3. Materials and Methods

### 3.1. Materials and General Techniques

The preparation and handling of the iron(II) and manganese(II) complexes were performed using standard Schlenk tube techniques under an argon atmosphere. Deuterated solvents CDCl_3_-*d*_1_ and CD_3_OD-*d*_4_ were obtained from Cambridge Isotope Laboratories, Inc. (Tewksbury, MA, USA). Heptane and tetrahydrofuran (thf) were carefully purified by refluxing and distilling under an argon atmosphere over sodium benzophenone ketyl. Ultra-dry solvents such as MeOH and CH_3_COOEt were purchased from Fujifilm Wako Chemicals, Corp. (Tokyo, Japan), and deoxygenated by purging with argon gas. Other reagents were commercially available and used without further purification. 2,6-Bis(5-isopropyl-1*H*-pyrazol-3-yl)pyridine (**L**) was prepared by a published method [19]. The purity of the obtained ligand **L** was checked by ^1^H-NMR spectroscopy in CDCl_3_-*d*_1_.

### 3.2. Measurements

Elemental analyses (C, H, N) were performed by the Open Facility Center for Research at Ibaraki University. IR (4000–400 cm^−1^) spectra were recorded on KBr pellets using a JASCO FT/IR-6300 spectrophotometer under ambient conditions (JASCO, Tokyo, Japan). Far-IR spectra (600–200 cm^−1^) were recorded as CsI pellets using a JASCO FT/IR 6700 spectrophotometer under vacuum conditions (JASCO, Tokyo, Japan). Raman spectra (4000–200 cm^−1^) were measured as neat powders on a JASCO RFT600 spectrophotometer with a YAG laser 600 mW (JASCO, Tokyo, Japan). Abbreviations used in the description of vibration data are as follows: vs, very strong; s, strong; m, medium; w, weak. ^1^H-NMR (500 MHz) spectra were obtained on a Bruker AVANCE III-500 NMR spectrometer at room temperature (298 K) in CDCl_3_-*d*_1_ or CD_3_OD-*d*_4_ (Bruker Japan, Yokohama, Japan). ^1^H chemical shifts were reported as *δ* values, referenced to residual solvent peaks (7.26 ppm and 3.31 ppm, respectively). UV-Vis absorption spectra in methanol under ambient conditions in the 220–1020 nm range were recorded with a JASCO V-570 spectrophotometer (JASCO, Tokyo, Japan). Diffuse reflectance (DR) spectra were obtained in the 200–1300 nm range with a JASCO V-570 spectrophotometer equipped with an integrating sphere apparatus (JASCO ISN-470) (JASCO, Tokyo, Japan).

### 3.3. Preparation of Complexes

[FeCl_2_(L)]

To a MeOH (5 mL) solution of anhydrous FeCl_2_ (46.7 mg, 0.368 mmol) in a 50 mL Schlenk tube was added dropwise a MeOH solution (10 mL) of **L** (105.1 mg, 0.356 mmol). After stirring for 3 h at RT (room temperature), the solvent was evaporated under reduced pressure and afforded an orange powder. Recrystallization from MeOH/CH_3_COOEt gave yellow crystals of [FeCl_2_(**L**)]. Yield: 74% (110.9 mg, 0.264 mmol).

Anal. Calcd for C_17_H_21_Cl_2_FeN_5_: C 48.37, H 5.01, N 16.59. Found: C 48.50, H 4.65, N 16.32.

IR (KBr) *ν*/cm^−1^: 3459w *ν*(O–H), 3182s *ν*(N–H), 3096m *ν*(C–H), 3066m *ν*(C–H), 2969s *ν*(C–H), 2934m *ν*(C–H), 2875m *ν*(C–H), 1610s, 1573s, 1509s, 1454s, 1280s, 1010s, 797s. Far–IR (CsI) *ν*/cm^−1^: 650m *ν*(Fe–N), 376w, 295s br *ν*(Fe–Cl), 261s br sh *ν*(Fe–Cl), 176 m. ^1^H-NMR (CD_3_OD-*d*_4_) *δ*/ppm (assignment): 57.43 (s, 2H, 4–pz*H*), {57.14 (s, 2H, 4–pz*H*)}, {49.72(s, 2H, 3,5–py*H*)}, 46.27(s, 2H, 3,5–py*H*), {22.82 (s, 1H, 4–py*H*)}, {13.79 (s, 2H, C*H*(CH_3_)_2_)}, 9.26 (s, 2H, C*H*(CH_3_)_2_), 3.76 (s, 12H, CH(C*H*_3_)_2_), 2.26 (s, 1H, 4–py*H*), {0.428 (s, 12H, CH(C*H*_3_)_2_)}. Values in parentheses (i.e., {}) are for the bis-chelate complex [Fe(**L**)_2_](FeCl_4_). UV-Vis (solution, MeOH) *λ*_max_/nm (molar absorptivity coefficient/M^−1^cm^−1^): 235 (17770), 253 (15670), 315 (9360), 461 (740). Diffuse reflectance (solid, neat) *λ*_max_/nm: 342, 472sh, 1116.

[Fe(L)_2_](PF_6_)_2_

To a MeOH (6 mL) solution of anhydrous FeCl_2_ (19.7 mg, 0.155 mmol) in a 50 mL Schlenk tube was added dropwise a MeOH solution (10 mL) of **L** (89.6 mg, 0.303 mmol). To the reaction mixture was added dropwise a MeOH solution (10 mL) of AgPF_6_ (83.3 mg, 0.329 mmol). After stirring for 4 h at RT, the solvent was evaporated under reduced pressure and afforded a brown-orange powder. Recrystallization from thf/heptane gave brown crystals of [Fe(**L**)_2_](PF_6_). Yield: 48% (67.5 mg, 0.072 mmol).

Anal. Calcd for C_34_H_42_F_12_FeN_10_P_2_·1/2(H_2_O + thf): C 44.05, H 4.83, N 14.27. Found: C 43.85, H 4.97, N 13.91. IR (KBr) *ν*/cm^−1^: 3651w *ν*(O–H), 3670w, 3371s *ν*(N–H), 3144w *ν*(C–H), 2972s *ν*(C–H), 2939m *ν*(C–H), 2879m *ν*(C–H), 1617s, 1577s, 1461s, 1350m, 1290m, 1018m*s*, 847vs *ν*(P–F), 557s *ν*(P–F). Far–IR (CsI) *ν*/cm^−1^: 651 w *ν*(Fe–N), 558 s *ν*(P–F), 371w, 197m. ^1^H-NMR (CD_3_OD-*d*_4_) *δ*/ppm (assignment): 57.61 (s, 4H, 4–pz*H*), 50.16 (s, 4H, 3,5–py*H*), 23.22 (s, 2H, 4–py*H*), 14.12 (s, 4H, C*H*(CH_3_)_2_), 0.70 (s, 24H, CH(C*H*_3_)_2_). UV-Vis (solution, MeOH) *λ*_max_/nm (molar absorptivity coefficient/M^−1^cm^−1^): 235 (40000), 252 (sh, 35020), 316 (19590), 462 (1490). Diffuse reflectance (solid, neat) *λ*_max_/nm: 262, 316, 396sh, 460, 826, 1098.

[MnCl_2_(L)]

To a MeOH (5 mL) solution of MnCl_2_∙4H_2_O (70.4 mg, 0.356 mmol) in a 50 mL Schlenk tube was added dropwise a MeOH solution (10 mL) of **L** (103.4 mg, 0.350 mmol). After stirring for 3 h at RT, the solvent was evaporated under reduced pressure and afforded a pale-yellow powder. Recrystallization from MeOH/CH_3_COOEt gave [MnCl_2_(bipPy)] as pale-yellow crystals. Yield: 72% (105.8 mg, 0.251 mmol).

Anal. Calcd for C_17_H_21_N_5_MnCl_2_·1/4(H_2_O): C 47.96, H 5.09, N 16.45. Found: C 47.96, H 4.99, N 16.38. IR (KBr) *ν*/cm^−1^: 3480sh *ν*(O–H), 3167s *ν*(N–H), 3098m *ν*(C–H), 3065m *ν*(C–H), 2970s *ν*(C–H), 29356m *ν*(C–H), 2873m *ν*(C–H), 1609s, 1573s, 1280s, 1012s, 797s. Far–IR (CsI) *ν*/cm^−1^: 668m *ν*(Mn–N), 553w, 501w, 302vs *ν*(Mn–Cl), 262vs *ν*(Mn–Cl), 182 m. Raman (neat solid) *ν*/cm^−1^: 3136w *ν*(N–H), 3072w *ν*(C–H), 2975m *ν*(C–H), 2889m *ν*(–CH_3_), 1611s 1575s, 1515s, 1511s, 1454s, 1419s, 1015s, 977m, 258w *ν*(Mn–Cl). UV-Vis (solution, MeOH) *λ*_max_/nm (molar absorptivity coefficient/M^−1^cm^−1^): 237 (19920), 255 (18080), 317 (10800). Diffuse reflectance (solid, neat) *λ*_max_/nm: 266, 328, 392sh, 416sh.

### 3.4. X-Ray Crystal Structure Determination

Crystal data and refinement parameters for the three complexes, [FeCl_2_(**L**)]·2(MeOH), [Fe(**L**)_2_](PF_6_)_2_·5(thf), and [MnCl_2_(**L**)]·2(MeOH), are given in Table S1. All crystallographic data have been deposited at the CCDC, 12 Union Road, Cambridge CB2 1EZ, UK, and copies can be obtained on request, free of charge, by quoting the publication citation and the deposition numbers: 2486924–2486927.

Diffraction data were measured on a Rigaku XtaLAB P200 diffractometer using multi-layer mirror monochromated Mo K*α* radiation (*λ* = 0.71075 Å) at a low temperature (Rigaku Oxford Diffraction, Oxfordshire, UK). A crystal of suitable size and quality was coated with Paratone N oil and mounted on a Dual-Thickness MicroLoop LD (200 μM) (MiTeGen) (MiTeGen, New York, NY, USA). The unit cell parameters were determined using *CrystalClear* from 18 images [44]. The crystal-to-detector distance was ca. 45 mm. Data were collected using 0.5° intervals in *φ* and *ω* to a maximum 2*θ* value of 55.0° (6–55°). The highly redundant data sets were reduced using *CrysAlisPro* (Rigaku Oxford Diffraction, Oxfordshire, UK) [45]. An empirical absorption correction was applied for each complex. Structures were solved by direct methods (*SIR2008* [46]). The position of the metal(II) ions and their first coordination sphere were located from a direct method *E*-map; other non-hydrogen atoms were found in alternating-difference Fourier syntheses, and least-squares refinement cycles. During the final refinement cycles, the temperature factors were refined anisotropically. Refinement was carried out by a full matrix least-squares method on *F*^2^. All calculations were performed with the *CrystalStructure* [47] crystallographic software package, except for refinement, which was performed using *SHELXL* 2013 [48]. Hydrogen atoms were placed in calculated positions. A weighting scheme of the form *w* = 1/[*σ*^2^ (*Fo*^2^) + (*aP*)^2^ + *bP*], where *p* = (*F_o_*^2^ + 2*F_c_*^2^)/3, was applied toward the latter stages of each refinement. The solvent molecules (ethyl acetate for [FeCl_2_(**L**)]·2(MeOH) and [MnCl_2_(**L**)]·2(MeOH) and thf/methanol for [Fe(**L**)_2_](PF_6_)_2_·5(thf)) in all complexes were also disordered to be modeled properly; thus, the program SQUEEZE, a part of the PLATON package of crystallographic software [49], was used to calculate the solvent disorder area and remove its contribution to the overall intensity data. The relatively higher *R* values in [Fe(**L**)_2_](PF_6_)_2_·5(thf) were due to the highly disordered PF_6_^–^ counter ions.

## 4. Conclusions

[MCl_2_(**L**)]·2(MeOH) complexes with different central metals (M = Fe and Mn) were synthesized and compared with the corresponding copper(II) and zinc(II) complexes described in our previous paper [19]. The manganese(II), iron(II), and zinc(II) dichlorido complexes possessed a distorted trigonal bipyramidal structure, while the copper(II) dichlorido complex exhibited a square pyramidal structure with significant elongation at the axial position due to the Jahn–Teller effect. The N–H group increased solubility in polar solvents. Various hydrogen bonds between N–H groups and the coordinated halogenide ions and some lattice solvents were observed in the solid-state structures. On complexation, the positions of the H atoms on the pyrazole nitrogens shifted remotely to the pyridine from adjacent to the pyridine ring. Only iron(II) dichlorido complex [FeCl_2_(**L**)]·2(MeOH) was not stable in polar solvents such as MeOH and formed the bis-chelate complex [Fe(**L**)_2_](FeCl_4_). Therefore, [Fe(**L**)_2_](PF_6_)_2_·5(thf) was synthesized by the reaction of [FeCl_2_(**L**)]·2(MeOH) with AgPF_6_. The possibility of spin-crossover was investigated for [Fe(**L**)_2_](PF_6_)_2_·5(thf). Color changes following cooling in liquid nitrogen suggested that this was a possibility. However, variable-temperature single-crystal X-ray structural analysis and spectroscopic results did not support this hypothesis over the temperature range investigated. The average Fe–N_py_ distance (2.138(2) Å) and the average Fe–N_pz_ distance (2.194(3) Å) of [Fe(**L**)_2_](PF_6_)_2_·5(thf) at low temperatures are within the range for a high-spin-state Fe(II) ion.

The structure and properties of the complexes obtained in this study deepen our understanding of this ligand group and provide useful data for future applications using this ligand system.

## Figures and Tables

**Table 1:** Temperature-dependent bond distances and angles for bis-chelate [Fe(ligand)_2_]X_2_-type complexes with coplanar N3 ligands.

Complexes ^a^	Temp /K	*d* (Fe–N_centeral_) (Avg.)/Å	*d* (Fe–N_terminal_) (Avg.)/Å	∠ (N_centeral_–Fe– N_centeral_)/°	∠ (N_terminal_–Fe– N_terminal_) (Avg.)/° ^b^	Spin State	Ref
**Type b** ^a^							
Fe(**L**)_2_](PF_6_)_2_ (R_b_ = iPr)	223	2.140 (3)	2.203 (3)	170.21 (10)	147.4 (1)	high-spin	tw ^c^
	140	2.138 (2)	2.194 (3)	169.93 (9)	147.52 (9)	high-spin	tw ^c^
Fe(Lb1)_2_](ClO_4_)_2_ (R_b_ = Me)	150	1.955 (3)	2.009 (3)	178.63 (11)	158.16 (11)	low-spin	32
Fe(Lb1)_2_](BF_4_)_2_ (R_b_ = Me)	300	2.109 (5)	2.171 (3)	180	148.8 (2)	high-spin	34
	150	1.984 (6)	2.033 (4)	180	154.9 (2)	low-spin	34
Fe(Lb2)_2_](ClO_4_)_2_ (R_b_ = NH_2_)	150	2.1567 (12)	2.191 (2)	177.33 (6)	146.87 (5)	high-spin	30
Fe(Lb3)_2_](BF_4_)_2_ (R_b_ = HNC(O)tBu)	150	2.145 (2)	2.197 (2)	158.30 (4)	147.32 (4)	high-spin	30
**Type a** ^a^							
Fe(terpy)_2_](PF_6_)_2_ (R_a_ = H)	294	1.891 (5)	1.988 (6)	178.6 (3)	161.1 (3)	low-spin	38
Fe(La1)_2_](PF_6_)_2_ (R_a_ = Ph)	296	1.881 (2)	1.977 (2)	175.19 (7)	161.97 (7)	low-spin	33
Fe(La2)_2_](BF_6_)_2_ (X = Cl)	120	2.080 (3)	2.272 (3)	175.4 (2)	149.8 (2)	high-spin	26
Fe(La3)_2_](BF_6_)_2_ (X = Br)	100	2.075 (5)	2.305 (5)	168.4 (2)	149.8 (2)	high-spin	26
**Type c** ^a^							
Fe(Lc1)_2_](ClO_4_)_2_ (R_c_ = H)	290	2.126 (2)	2.184 (3)	173.15 (10)	146.83 (9)	high-spin	36
Fe(Lc1)_2_](ClO_4_)_2_ (R_c_ = H)	240	1.899 (3)	1.976 (4)	178.25 (18)	160.04 (13)	low-spin	36
Fe(Lc2)_2_](ClO_4_)_2_ (R_c_ = Mes)	150	1.894 (2)	1.997 (2)	178.98 (8)	168.62 (8)	low-spin	35

^a^ Type was related to Figure 1. Lb1 = 2,6-bis(5-methyl-1*H*-pyrazol-3-yl)pyridine; Lb2 = 2,6-bis(5-amino-1*H*-pyrazol-3-yl)pyridine; Lb3 = 2,6-bis(5-{tertiary-butyl-amido}-1*H*-pyrazol-3-yl)pyridine; La1 = 4′-phenyl-2,2′:6′,2″-terpyridine; La2 = 6,6″-dichloro-2,2′:6′,2″-terpyridine; La3 = La2 = 6,6″-dibromo-2,2′:6′,2″-terpyridine; Lc1 = 2,6-di(pyrazol-1-yl)pyridine; Lc2 = 2,6-bis-{3-[2,4,6-trimethylphenyl]pyrazol-1-yl}pyridine; ^b^ face-to face distances. ^c^ tw denotes “this work”.

## Data Availability

The crystallographic data are available from the Cambridge Crystallographic Data Centre (CCDC).

## Supplementary Materials

The following supporting information can be downloaded at https://www.mdpi.com/article/10.3390/molecules30204128/s1, Figure S1: ORTEP view of [FeCl_2_(L)]·2(MeOH); Figure S2: Packing view of [FeCl_2_(**L**)]·2(MeOH); Figure S3: ORTEP view of [Fe(**L**)_2_](PF_6_)_2_·5(thf) (−133 °C); Figure S4: ORTEP view of [Fe(**L**)_2_](PF_6_)_2_·5(thf) (−50 °C); Figure S5: ORTEP view of cation part of [Fe(**L**)_2_](PF_6_)_2_·5(thf) (−50 °C); Figure S6: ORTEP view of [MnCl_2_(L)]·2(MeOH); Figure S7: Packing view of [MnCl_2_(L)]·2(MeOH); Figure S8: IR spectra of L and iron(II) and manganese(II) complexes; Figure S9: Raman spectra of **L** and [MnCl_2_(**L**)]; Figure S10: ^1^H-NMR spectrum of [FeCl_2_(**L**)]·2(MeOH) in CD_3_OD; Figure S11: ^1^H-NMR spectrum of [FeCl_2_(**L**)]·2(MeOH) in CD_3_OD after 48 h; Figure S12: Time-dependent ^1^H-NMR spectral changes of [FeCl_2_(**L**)]·2(MeOH); Figure S13: UV-Vis absorption spectra of [FeCl_2_(**L**)]·2(MeOH) and [Fe(**L**)_2_](PF_6_)_2_·5(thf); Table S1: Summary of crystallographic data for iron(II) and manganese(II) complexes.

## Abbreviations

The following abbreviations are used in this manuscript:

bispzHpy	2,6-bis(1*H*-pyrazol-3-yl)pyridine
bispzpy	2,6-bis(*N*-pyrazol)pyridine
CCDC	Cambridge Crystallographic Data Centre
DR	Diffuse Reflectance
IR	Infrared
MeOH	Methanol
NMR	Nuclear Magnetic Resonance
ORTEP	Oak Ridge Thermal Ellipsoid Program
OTf^−^	Trifluoromethanesulfonate Anion
py	Pyridine
pz	Pyrazole
terpy	2,2′:6′,2″-terpyridine
thf	Tetrahydrofuran
UV-Vis	Ultraviolet–Visible
