# Supplementary material for: Iron(II) and Manganese(II) Coordination Chemistry Ligated by Coplanar Tridentate Nitrogen-Donor Ligand, 2,6-bis(5-isopropyl-1H-pyrazol-3-yl)pyridine
Source: Molecules. 2025 Oct 19;30(20):4128. doi: 10.3390/molecules30204128 (PMC12566300; doi:10.3390/molecules30204128)
Supplement: Supplementary file 1 [file molecules-30-04128-s001.zip › SM Fe(II) and Mn(II) with 2,6-bis(5-isopropyl-1H-pyrazol-3-yl)pyridine.pdf]

SupplementaryMaterials:

Iron(II) and manganese(II) coordination chemistry ligated by coplanar tridentate ligand, 2,6-bis(5-isopropyl-1*H*-pyrazol-3-yl)pyridine

Kiyoshi Fujisawa*, Yurika Minakawa, and David James Young

Content

**Figure S1**	ORTEP view of [$FeCl_2$(**L**)]·2(MeOH).	S1
**Figure S2**	Packing view of [$FeCl_2$(**L**)]·2(MeOH).	S2
**Figure S3**	ORTEP view of the bis-chelate iron(II) complex [Fe(**L**)$_2$](PF_6)$_2$·5(thf) (–133 °C).	S3
**Figure S4**	ORTEP view of the bis-chelate iron(II) complex [Fe(**L**)$_2$](PF_6)$_2$·5(thf) (–50 °C).	S4
**Figure S5**	ORTEP view of cation part of [Fe(**L**)$_2$](PF_6)$_2$·5(thf) (–50 °C).	S5
**Figure S6**	ORTEP view of [$MnCl_2$(**L**)]·2(MeOH).	S6
**Figure S7**	Packing view of [$MnCl_2$(**L**)]·2(MeOH).	S7
**Figure S8**	IR spectra of **L** and iron(II) and manganese(II) complexes.	S8
**Figure S9**	Raman spectra of **L** and [$MnCl_2$(**L**)]·2(MeOH).	S9
**Figure S10**	^{1}H-NMR spectrum of [$FeCl_2$(**L**)]·2(MeOH) in CD_3OD.	S10
**Figure S11**	^{1}H-NMR spectrum of [$FeCl_2$(**L**)]·2(MeOH) in CD_3OD after 48 hours.	S10
**Figure S12**	Time dependent ^{1}H-NMR spectral changes of [$FeCl_2$(**L**)]·2(MeOH).	S11
**Figure S13**	UV–Vis absorption spectra of [$FeCl_2$(**L**)]·2(MeOH) and [Fe(**L**)$_2$](PF_6)$_2$·5(thf).	S12
**Table S1**	Summary of crystallographic data for iron(II) and manganese(II) complexes.	S13

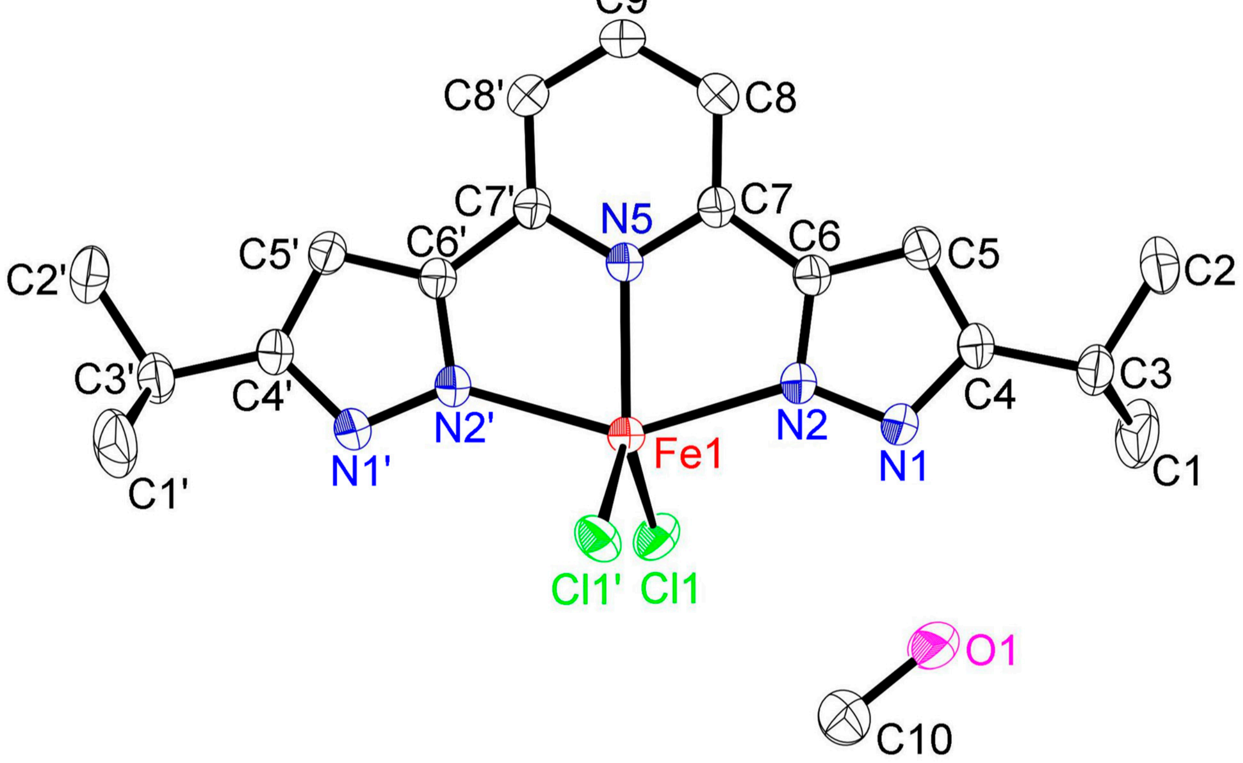

Figure S1. ORTEP view of $[FeCl_2(\mathbf{L})]\cdot 2(MeOH)$ showing 50% displacement ellipsoids and the atom-labelling scheme. Hydrogen atoms are omitted for clarity. Symmetry operators, –X+1/2, Y, –Z.

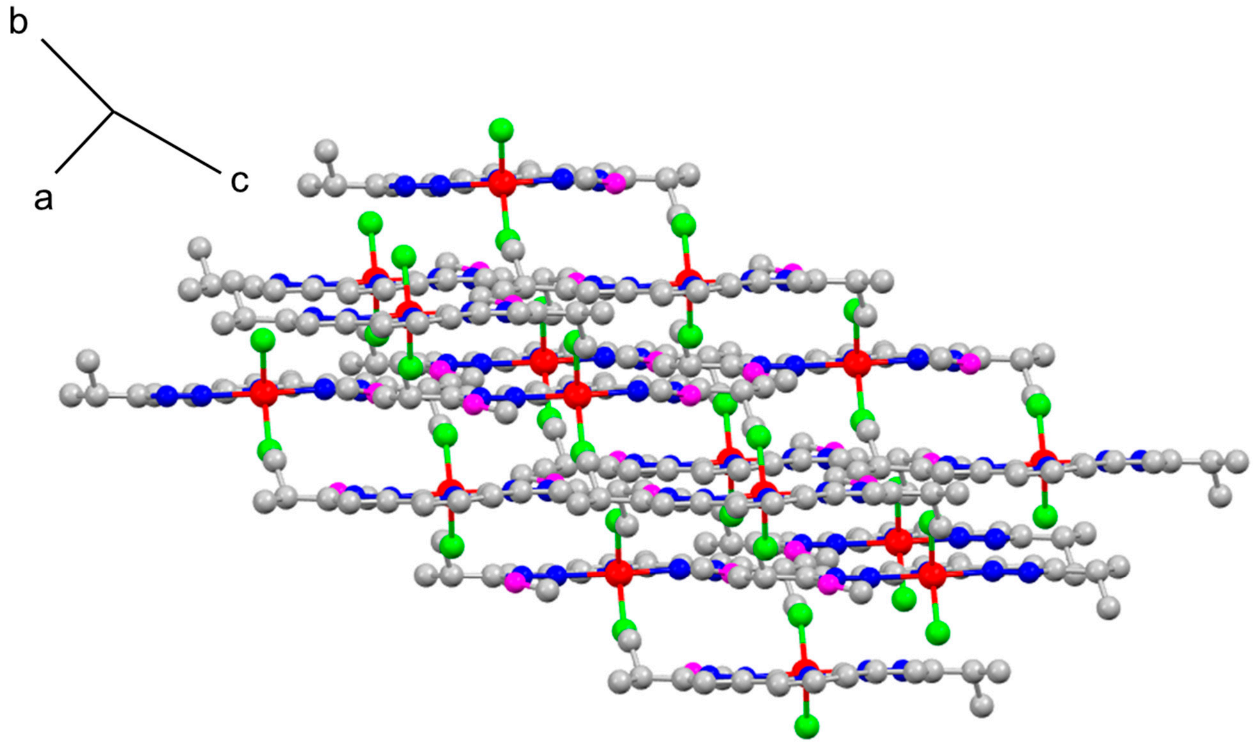

Figure S2. Packing view of $[FeCl_2(\mathbf{L})]\cdot 2(MeOH)$. Hydrogen atoms are omitted for clarity. Color: iron, red; green, chlorine, blue, nitrogen; magenta, oxygen; grey, carbon.

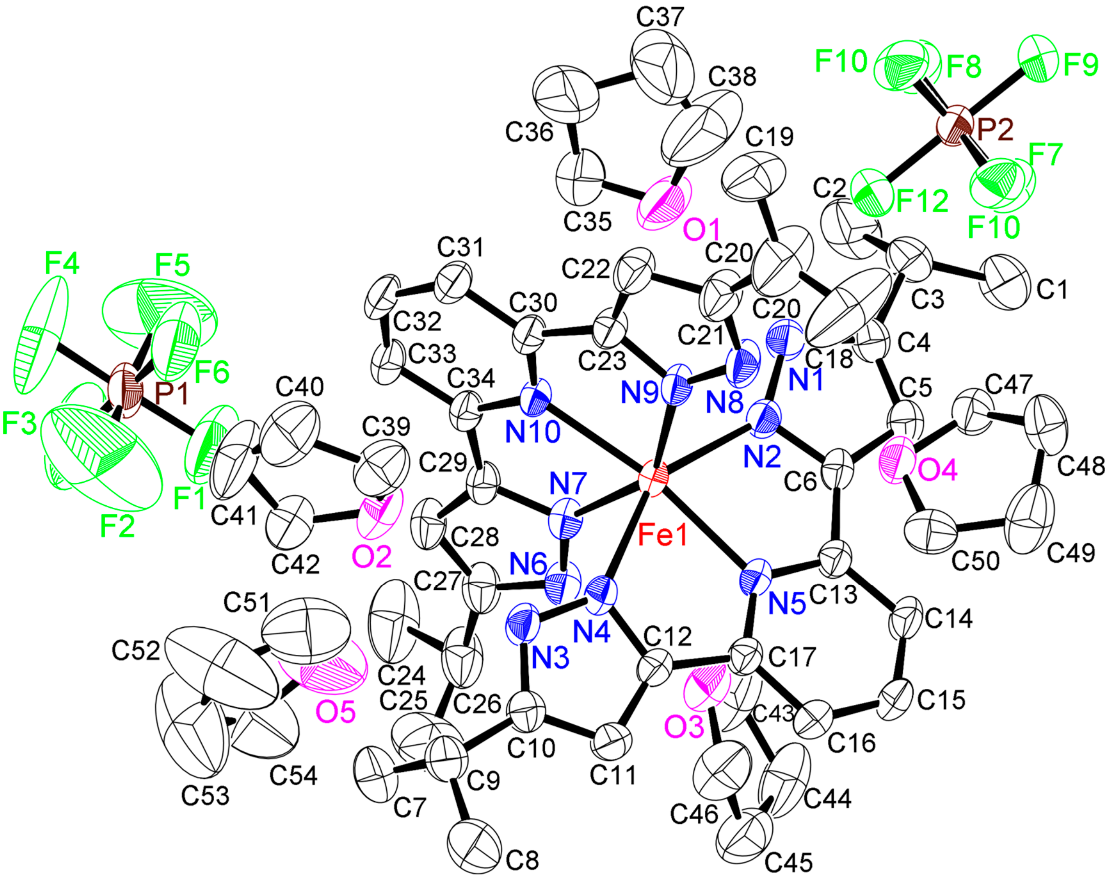

Figure S3. ORTEP view of cation part of [Fe(**L**)$_2$](PF$_6$)$_2$·5(thf) (–133 °C) showing 50% displacement ellipsoids with the atom-labelling scheme. Hydrogen atoms are omitted for clarity.

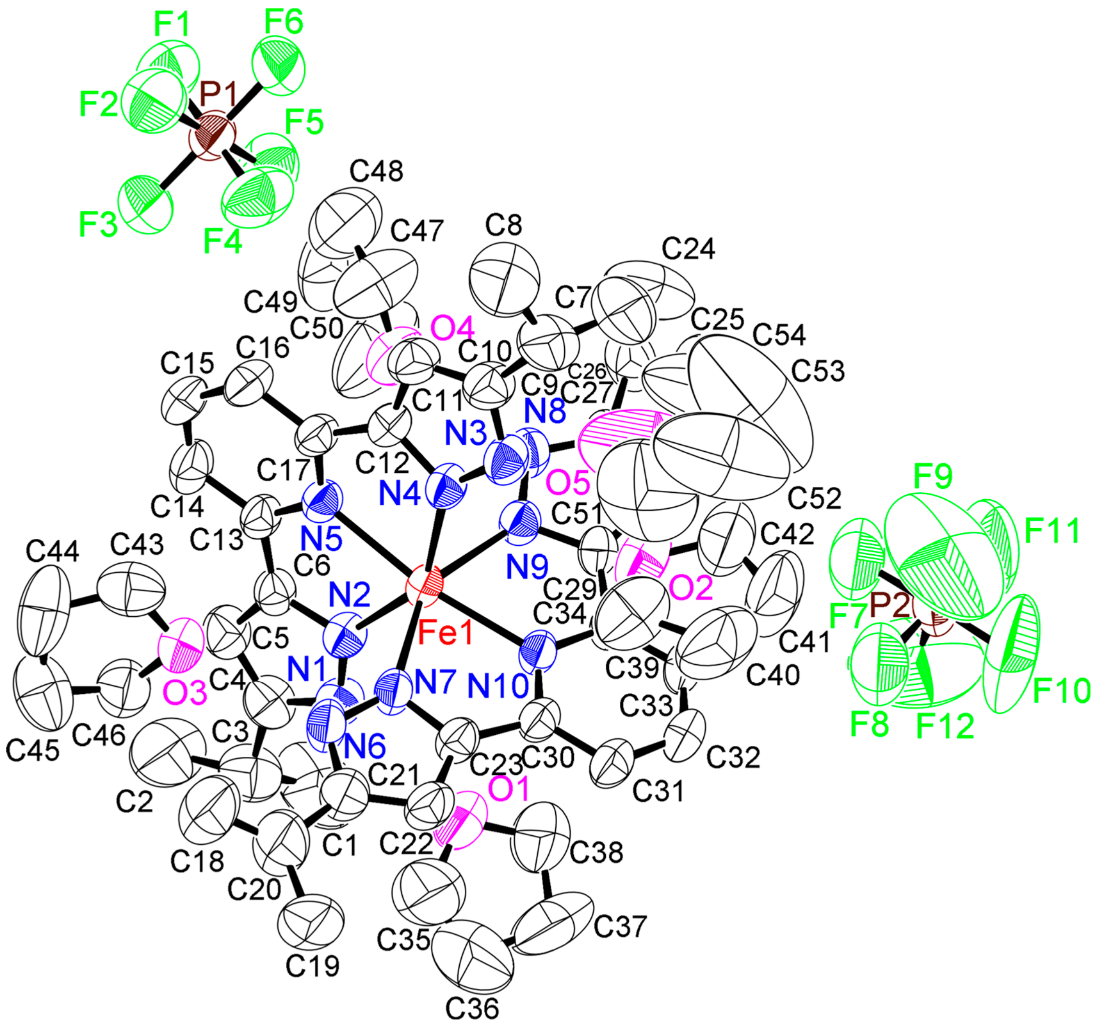

Figure S4. ORTEP view of [Fe(**L**)$_2$](PF$_6$)$_2$·5(thf) (–50 °C) showing 50% displacement ellipsoids with the atom-labelling scheme. Hydrogen atoms are omitted for clarity. Important bond lengths (Å) and angles (°) around iron(II) center: Fe1–N2, 2.184(3); Fe1–N4, 2.221(3); Fe1–N5, 2.143(3); Fe1–N7, 2.195(3); Fe1–N9, 2.211(3); Fe1–N10, 2.136(3); N2–Fe1–N4, 147.63(11); N2–Fe1–N5, 74.04(10); N2–Fe1–N7, 90.54(10); N2–Fe1–N9, 99.94(10); N2–Fe1–N10, 114.75(10); N4–Fe1–N5, 73.58(10); N4–Fe1–N7, 101.45(10); N4–Fe1–N9, 86.17(10); N4–Fe1–N10, 97.51(10); N5–Fe1–N7, 111.46(10); N5–Fe1–N9, 101.35(10); N5–Fe1–N10, 170.21(10); N7–Fe1–N9, 147.16(10); N7–Fe1–N10, 73.81(10); N9–Fe1–N10, 73.53(10).

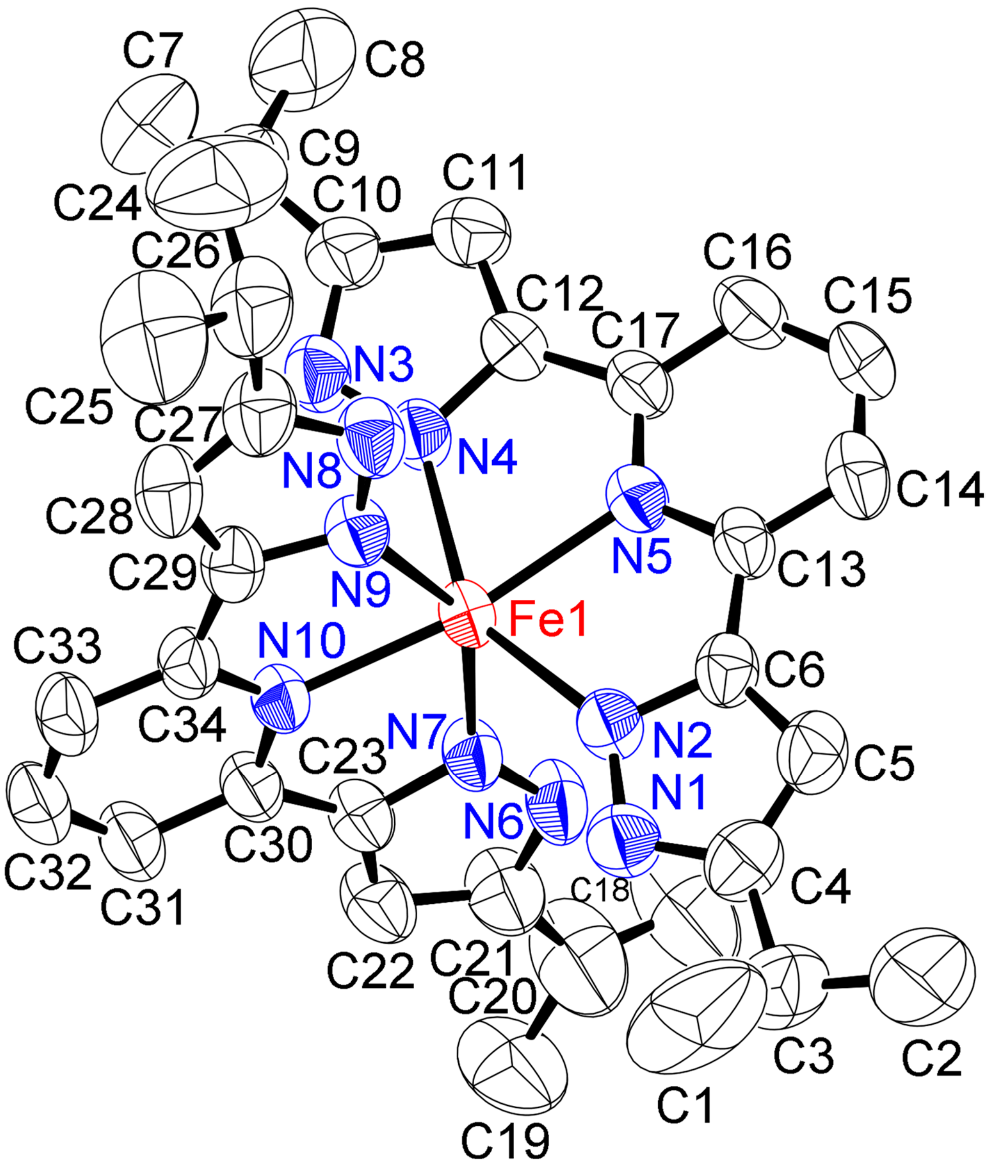

Figure S5. ORTEP view of cation part of the bis-chelate iron(II) complex [Fe(**L**)$_2$](PF$_6$)$_2$·5(thf) (–50 °C) (50% displacement ellipsoids) with the atom-labelling scheme. Hydrogen atoms, counter ions (PF$_6^-$), and thf molecules of solvates are omitted for clarity.

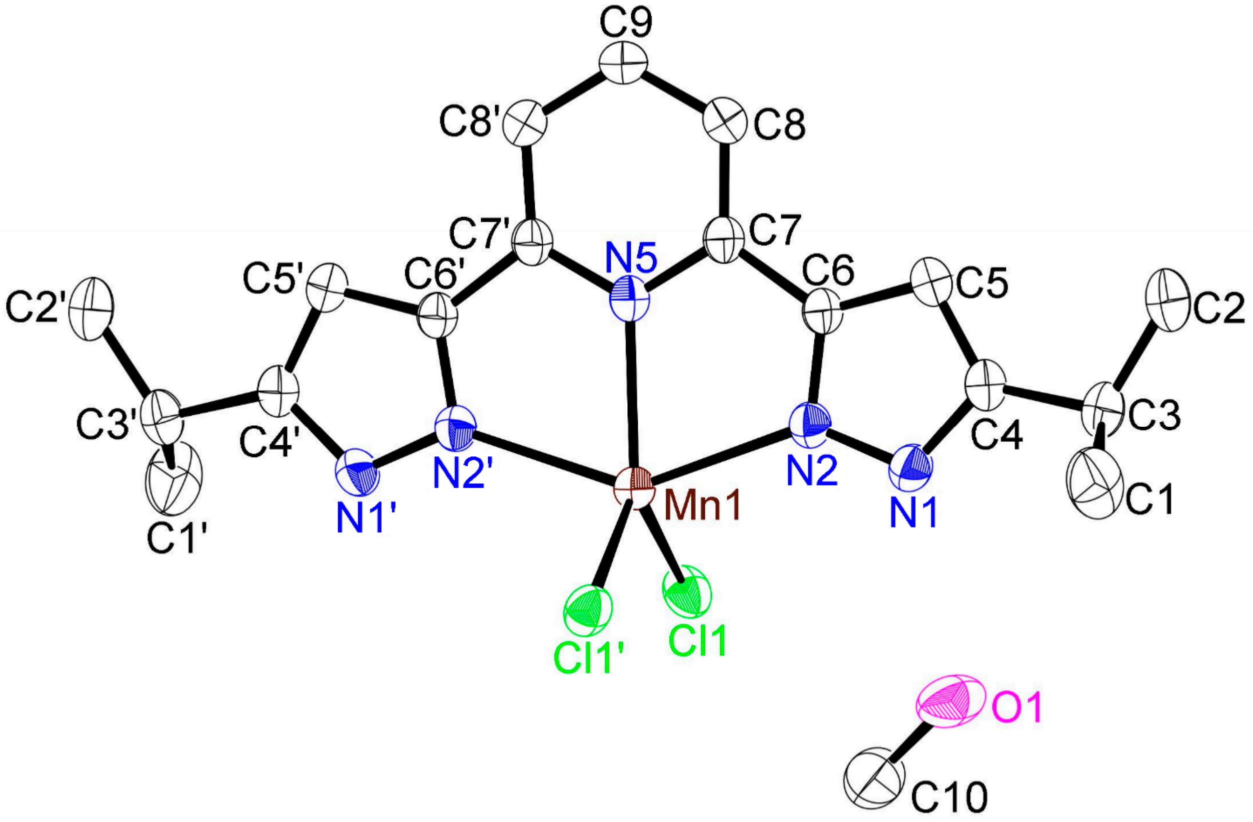

Figure S6. ORTEP view of $[MnCl_2(\mathbf{L})]\cdot 2(MeOH)$ showing 50% displacement ellipsoids and the atom-labelling scheme. Hydrogen atoms are omitted for clarity. Symmetry operators, –X+1/2, Y, –Z.

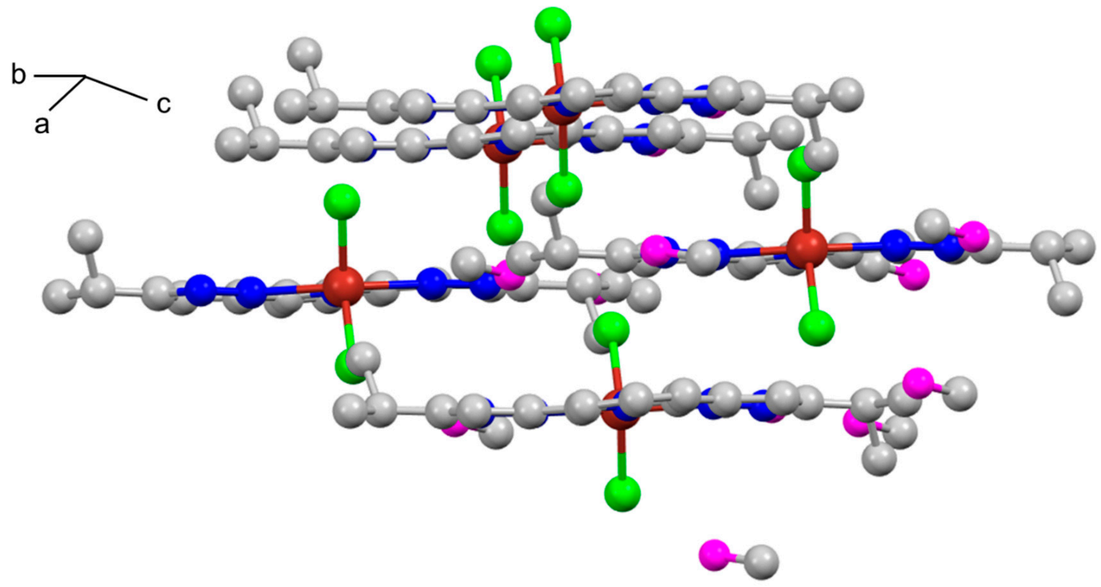

Figure S7. Packing view of [$MnCl_2$(**L**)]·2(MeOH). Hydrogen atoms are omitted for clarity. Color: manganese, brown; green, chlorine, blue, nitrogen; magenta, oxygen; grey, carbon.

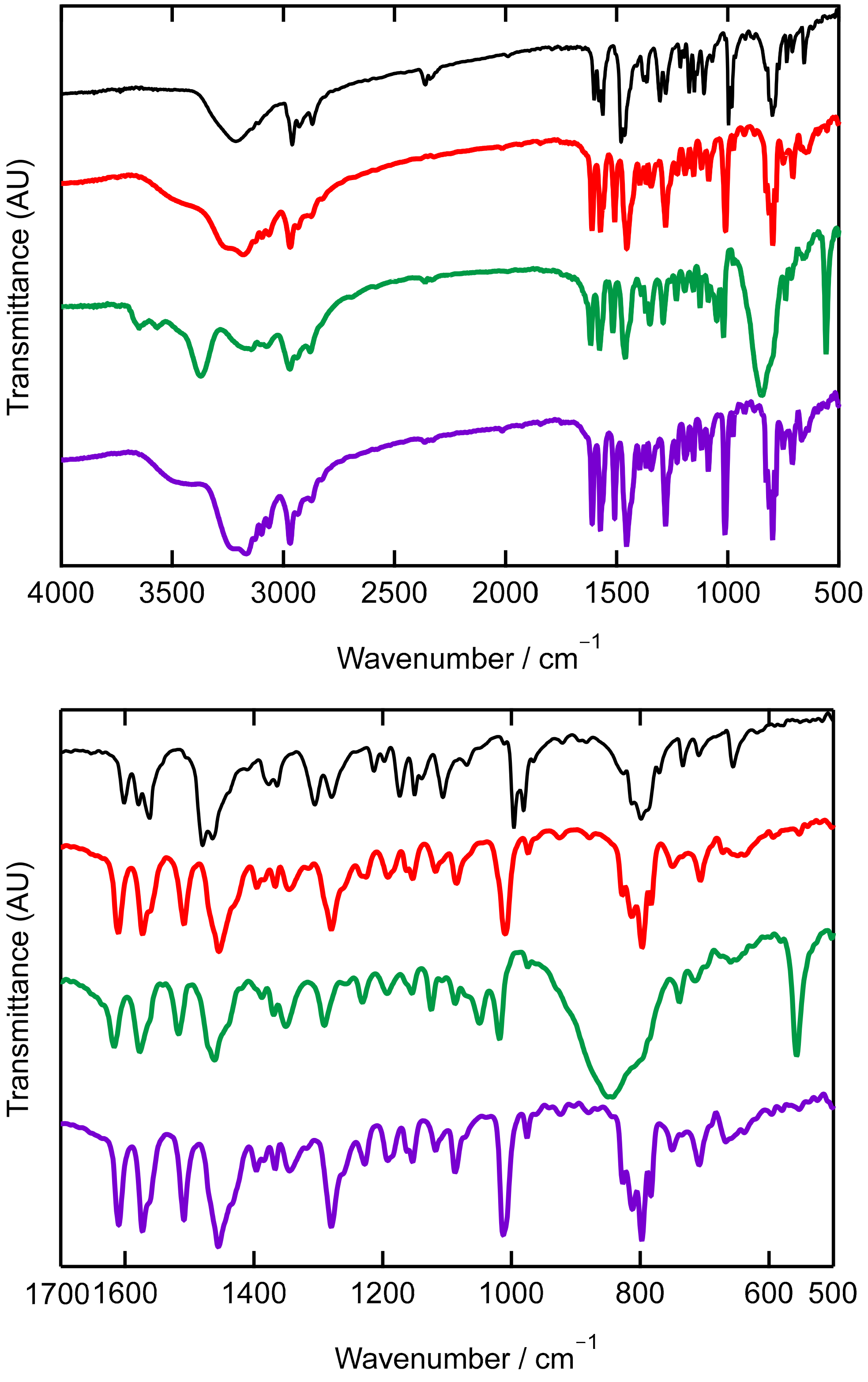

Figure S8. IR spectra of the ligand (**L**) (black trace), $[FeCl_2(\mathbf{L})]\cdot 2(MeOH)$ (red trace), $[Fe(\mathbf{L})_2](PF_6)_2\cdot 5(thf)$ (green trace), and $[MnCl_2(\mathbf{L})]\cdot 2(MeOH)$ (purple trace) in the range 4000 to 500 cm^{-1} (upper) and 1700 to 500 cm^{-1} (bottom).

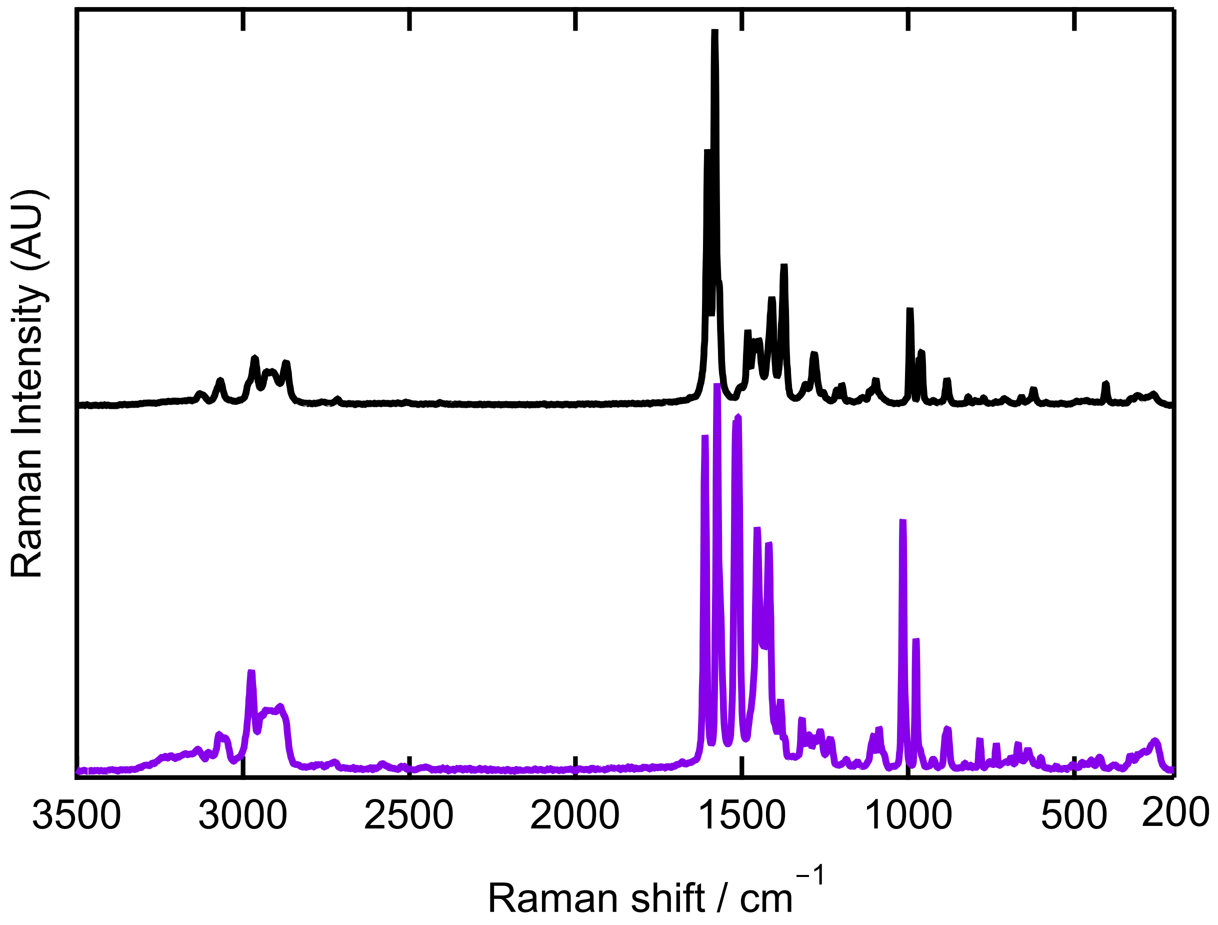

Figure S9. Raman spectra of the ligand **L** (black trace), and [$MnCl_2$(**L**)]·2(MeOH) (purple trace) in the range 3500 to 200 cm^{-1}.

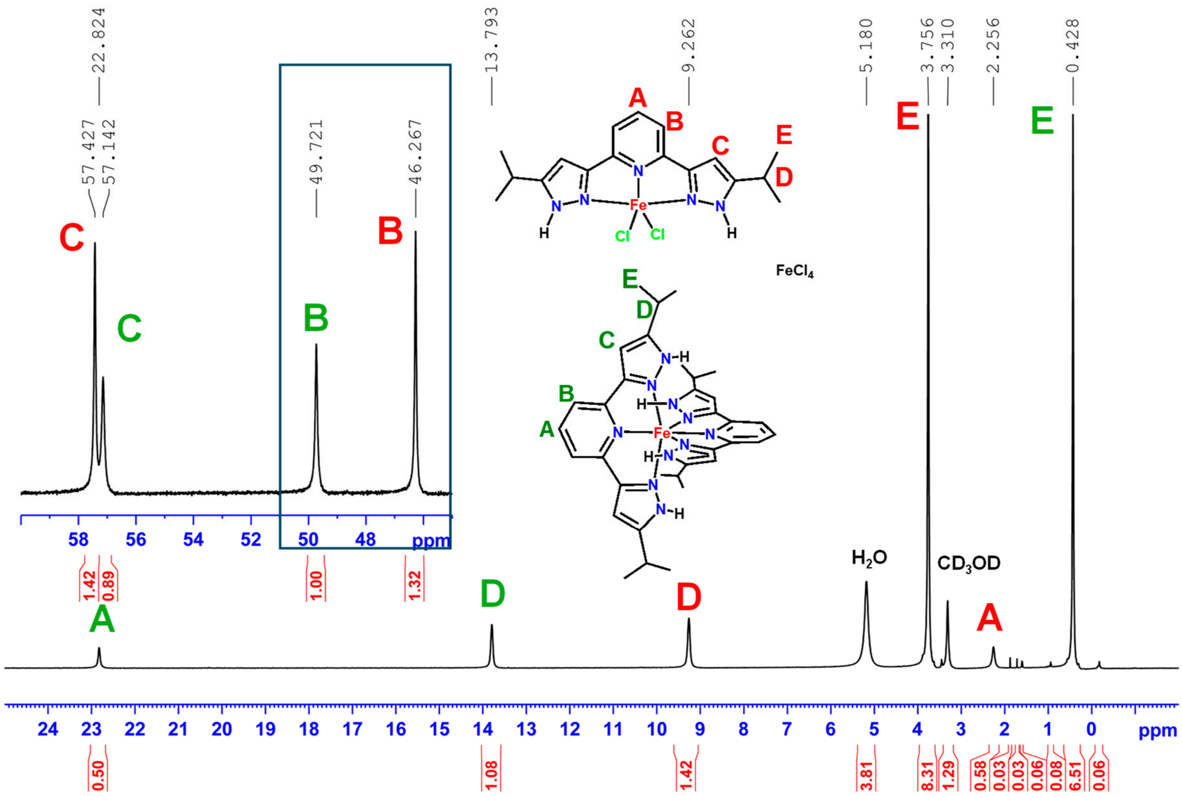

Figure S10. ^{1}H-NMR spectrum of $[FeCl_2(\mathbf{L})]\cdot 2(MeOH)$ in CD_3OD at room temperature at 0 min.

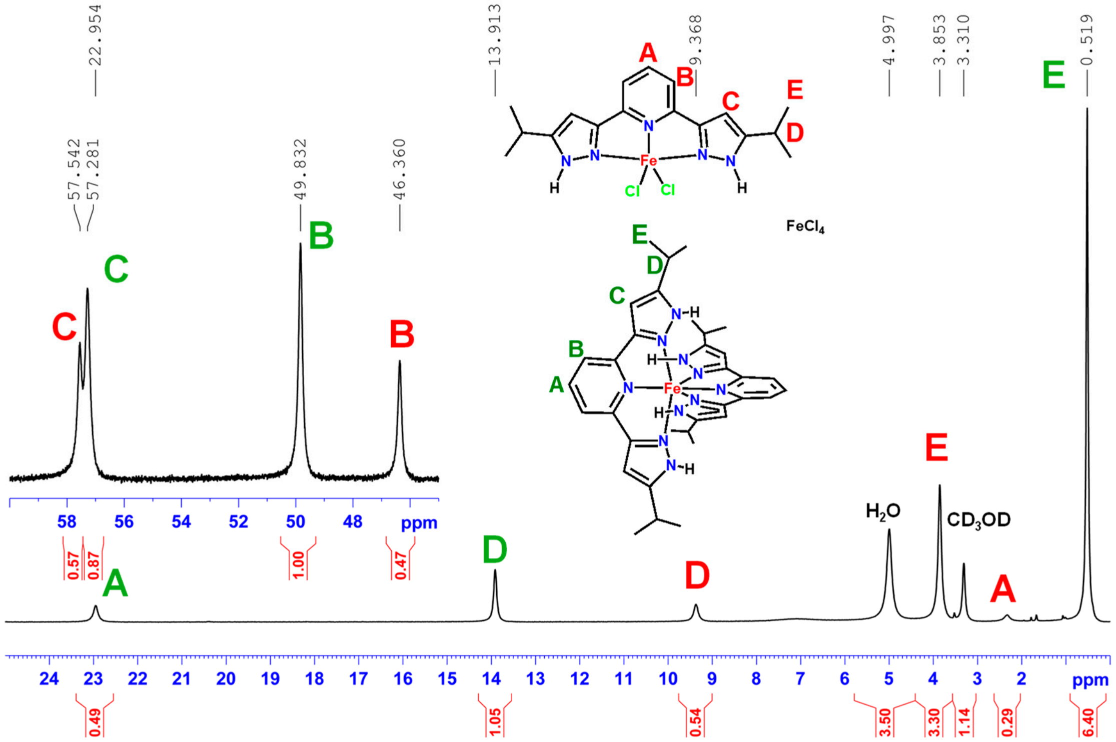

Figure S11. ^{1}H-NMR spectrum of $[FeCl_2(\mathbf{L})]\cdot 2(MeOH)$ in CD_3OD at room temperature at 48 hours after.

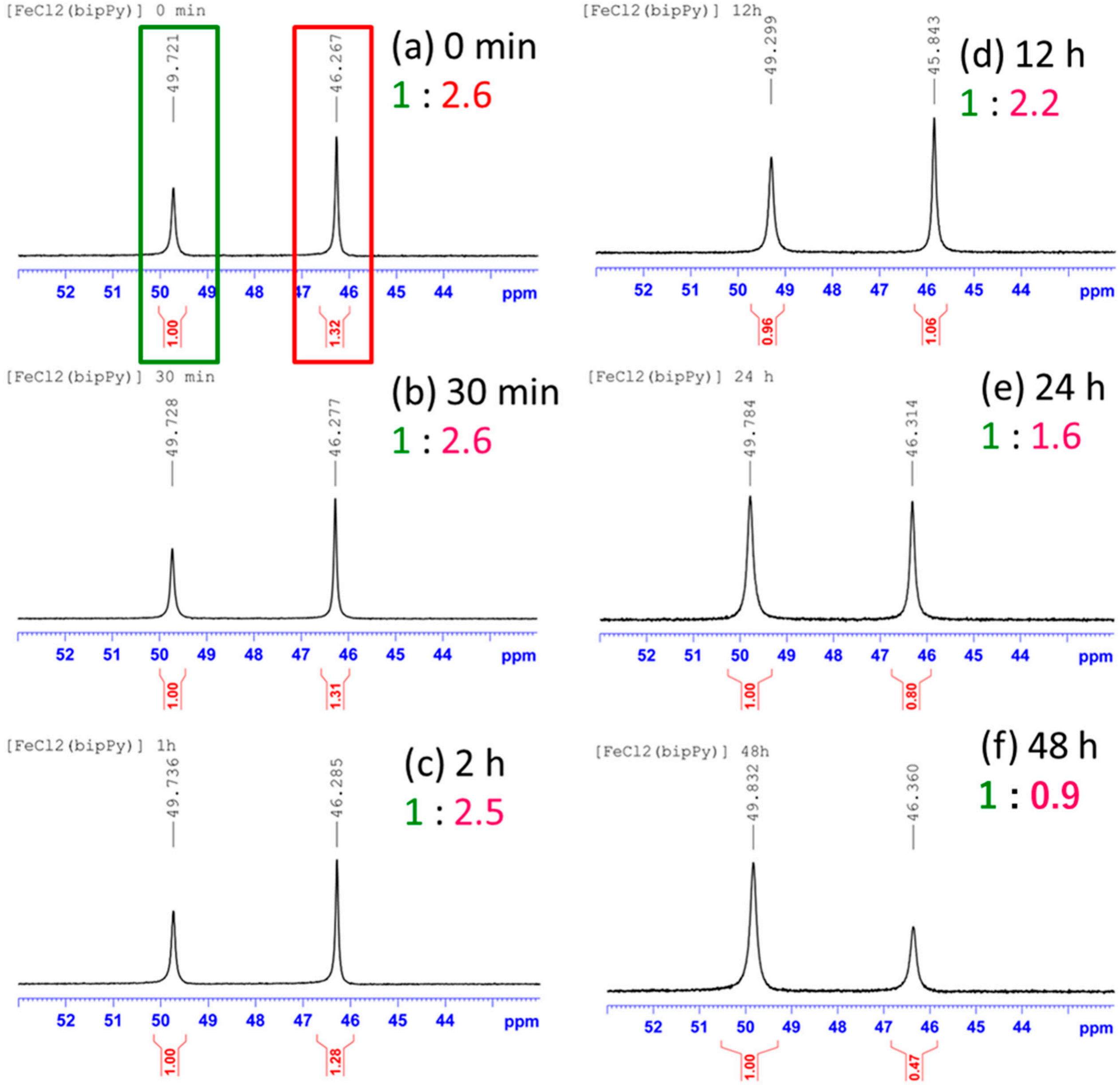

Figure S12. Time dependent ^{1}H-NMR spectral changes of $[FeCl_2(\mathbf{L})]\cdot 2(MeOH)$ to form $[Fe(\mathbf{L})_2](FeCl_4)$ in CD_3OD at room temperature at the pyrazole 4-proton region after (a) 0 min, (b) 30 min, (c) 2 h, (d) 12 h, (e) 24 h, and (f) 48 h after.

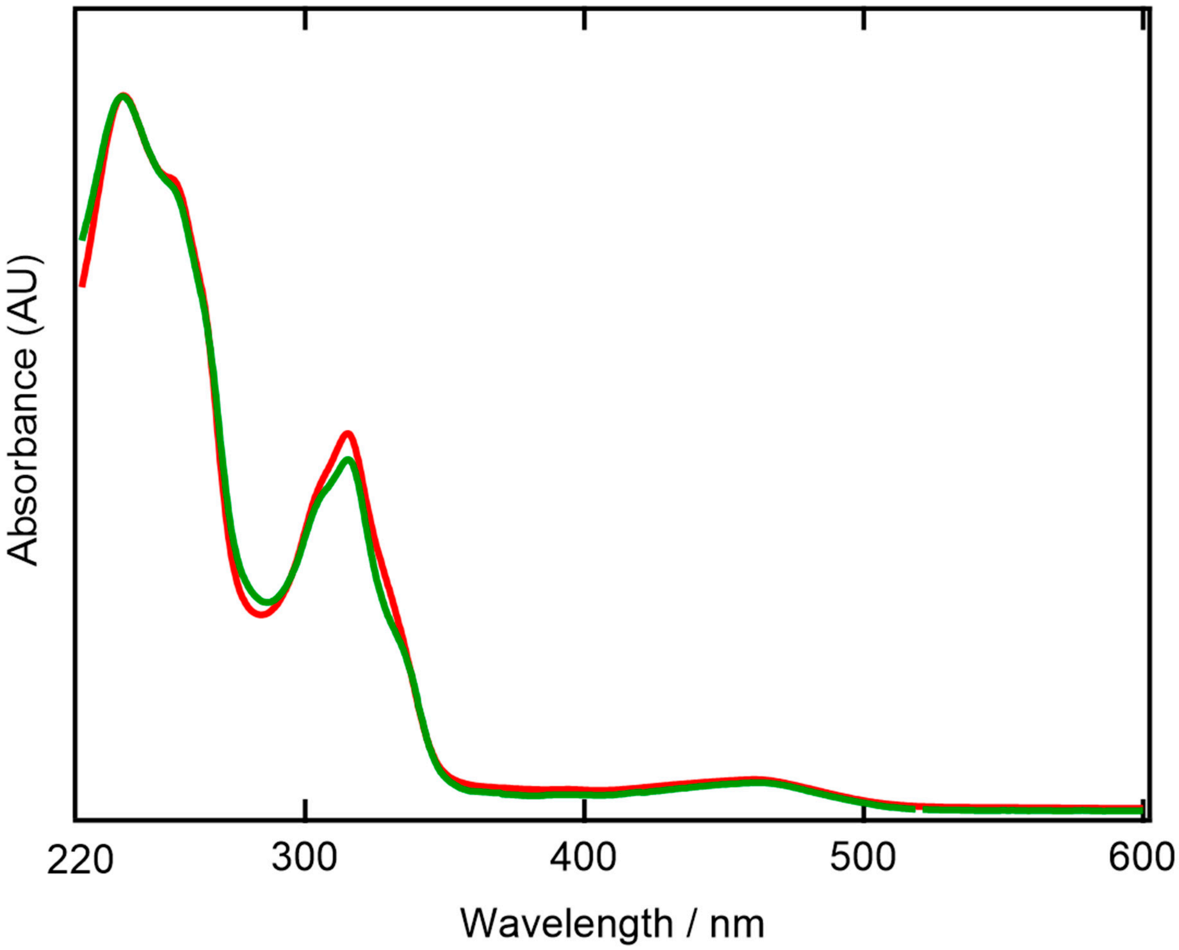

Figure S13. UV–Vis absorption spectra of $[FeCl_2(\mathbf{L})]\cdot2(MeOH)$ (red trace) and $[Fe(\mathbf{L})_2](PF_6)_2\cdot5(thf)$ (green trace) at room temperature in MeOH.

Table S1. Summary of crystallographic data for iron(II) and manganese(II) complexes.

Complexes	[FeCl$_2$(**L**)]·2(MeOH)	[Fe(**L**)$_2$](PF$_6$)·5(thf)	[Fe(**L**)$_2$](PF$_6$)·5(thf)	[MnCl$_2$(**L**)]·2(MeOH)
Formula	$C_{19}H_{29}Cl_2FeN_5O_2$	$C_{54}H_{82}F_{12}FeN_{10}O_5P_2$	$C_{54}H_{82}F_{12}FeN_{10}O_5P_2$	$C_{19}H_{29}Cl_2MnN_5O_2$
CCDC number	2486924	2686925	2486926	2486927
Temperature	–95±1	–133±1	–50±1	–95±1
Molecular weight	486.22	1297.08	1297.08	485.31
Crystal system	Monoclinic (*I*-centered)	Monoclinic (Primitive)	Monoclinic (Primitive)	Monoclinic (*I*-centered)
Space group	*I*2/*a* (#15)	$P2_1/c$ (#14)	$P2_1/c$ (#14)	*I*2/*a* (#15)
a /Å	11.13254(19)	21.9220(3)	22.0491(5)	11.0968(3)
b /Å	19.5519(2)	13.0779(3)	13.2593(3)	19.6141(4)
c /Å	13.9995(2)	24.7185(4)	24.9553(5)	14.0811(3)
β /°	111.6740(19)	91.4780(14)	91.532(2)	111.742(2)
V /Å^3	2831.73(8)	7084.3(2)	7293.2(3)	2846.78(12)
Z	4	4	4	4
D_{calc} /g cm^{-3}	1.140	1.216	1.1814	1.132
μ/cm^{-1}	7.402	3.361	3.265	6.706
Reflections meas.	46333	111555	116784	46526
Unique reflections	3262	16250	16734	3281
Observed data ($I \geq 2.0\sigma(I)$)	3093	12428	9165	3026
R (int)	0.0158	0.0341	0.0510	0.0538
No. parameters	138	757	757	135
R ($I > 2\sigma(I)$) [a]	0.0331	0.0745	0.0800	0.0420
R (◎ ll refls.) [a]	0.0343	0.0939	0.1338	0.0444
$wR2$ ($I > 2\sigma(I)$) [a]	0.1170	0.2158	0.2491	0.1373
wR2(◎ ll refls.) [a]	0.1177	0.2333	0.2846	0.1404
a; *b* in weighting scheme	0.0511; 4.0151	0.01324; 9.1961	0.1816; 0.0000	0.0887; 1.9066
Range of residual e-density peaks /eÅ^{-3}	–0.31 to 0.45	–0.62 to 1.11	–0.39 to 0.61	–0.51 to 0.69

[a] $R = \Sigma \mid |Fo| - |Fc| \mid / \Sigma \mid Fo \mid$;
$wR2 = [(\Sigma (w (|Fo|^2 - |Fc|^2)^2) / \Sigma w (Fo^2))^2]^{1/2}$, $w = 1/[\sigma^2(F_o^2) + (aP)^2 + bP]$, where $P = (F_o^2 + 2F_c^2)/3$.
